# The Rac-GEF Tiam1 controls integrin-dependent neutrophil responses

**DOI:** 10.3389/fimmu.2023.1223653

**Published:** 2023-11-21

**Authors:** Kirsti Hornigold, Martin J. Baker, Polly A. Machin, Stephen A. Chetwynd, Anna-Karin Johnsson, Chiara Pantarelli, Priota Islam, Melanie Stammers, Laraine Crossland, David Oxley, Hanneke Okkenhaug, Simon Walker, Rachael Walker, Anne Segonds-Pichon, Yoshinori Fukui, Angeliki Malliri, Heidi C. E. Welch

**Affiliations:** ^1^ Signalling Programme, Babraham Institute, Cambridge, United Kingdom; ^2^ Cell Signalling Group, Cancer Research UK Manchester Institute, University of Manchester, Macclesfield, United Kingdom; ^3^ Babraham Institute, Cambridge, United Kingdom; ^4^ Mass Spectrometry Facility, Babraham Institute, Cambridge, United Kingdom; ^5^ Imaging Facility, Babraham Institute, Cambridge, United Kingdom; ^6^ Flow Cytometry Facility, Babraham Institute, Cambridge, United Kingdom; ^7^ Bioinformatics Facility, Babraham Institute, Cambridge, United Kingdom; ^8^ Division of Immunogenetics, Department of Immunobiology and Neuroscience, Medical Institute of Bioregulation, Kyushu University, Fukuoka, Japan

**Keywords:** Tiam1, Rac, neutrophils, Rac-GEF, small GTPase, Prex1, Vav, Dock2

## Abstract

Rac GTPases are required for neutrophil adhesion and migration, and for the neutrophil effector responses that kill pathogens. These Rac-dependent functions are impaired when neutrophils lack the activators of Rac, Rac-GEFs from the Prex, Vav, and Dock families. In this study, we demonstrate that Tiam1 is also expressed in neutrophils, governing focal complexes, actin cytoskeletal dynamics, polarisation, and migration, in a manner depending on the integrin ligand to which the cells adhere. Tiam1 is dispensable for the generation of reactive oxygen species but mediates degranulation and NETs release in adherent neutrophils, as well as the killing of bacteria. *In vivo*, Tiam1 is required for neutrophil recruitment during aseptic peritonitis and for the clearance of *Streptococcus pneumoniae* during pulmonary infection. However, Tiam1 functions differently to other Rac-GEFs. Instead of promoting neutrophil adhesion to ICAM1 and stimulating β2 integrin activity as could be expected, Tiam1 restricts these processes. In accordance with these paradoxical inhibitory roles, Tiam1 limits the fMLP-stimulated activation of Rac1 and Rac2 in adherent neutrophils, rather than activating Rac as expected. Tiam1 promotes the expression of several regulators of small GTPases and cytoskeletal dynamics, including αPix, Psd4, Rasa3, and Tiam2. It also controls the association of Rasa3, and potentially αPix, Git2, Psd4, and 14-3-3ζ/δ, with Rac. We propose these latter roles of Tiam1 underlie its effects on Rac and β2 integrin activity and on cell responses. Hence, Tiam1 is a novel regulator of Rac-dependent neutrophil responses that functions differently to other known neutrophil Rac-GEFs.

## Introduction

Neutrophils are leukocytes of the innate immune system that provide the first line of defence against bacterial and fungal infections, as well as fulfilling important roles in inflammation. They are recruited from the blood stream into infected tissues where they phagocytose, degranulate, and generate reactive oxygen species (ROS) and neutrophil extracellular traps (NETs) to destroy invaded microorganisms (1–3).

Rac is a family of Rho-type small G proteins (GTPases) essential for neutrophil-mediated host defence. Three isoforms are expressed in neutrophils, the ubiquitous Rac1, hematopoietic Rac2, and RhoG, which is widely expressed but more distantly related. Collectively, these GTPases control the actomyosin cytoskeleton, enabling the formation of branched F-actin at the cell periphery which is required for adhesion, spreading, polarisation, and migration. By controlling the cytoskeleton, they also influence degranulation and phagocytosis. Additionally, active Rac2 is an essential component of the NADPH oxidase complex and therefore required for the production of ROS and NETs (4).

Like most small GTPases, Rac proteins are molecular switches, active in their GTP-bound form and inactive in their GDP-bound form (5). Their activation is catalysed by a large number of guanine-nucleotide exchange factors (GEFs), which remove bound GDP, allowing free cellular GTP to bind and induce a conformational change. The GTP-bound conformation enables Rac to interact with its effector proteins (6, 7), such as Irsp53 and Wave which signal to Arp2/3 to stimulate actin polymerisation (8).

Neutrophil Rac-GEFs include Prex, Vav, and Dock family proteins, whose roles have been identified using mouse genetics. *Prex1^–/–^* mouse neutrophils show decreased actin polymerisation, adhesion, migration speed, ROS production, and killing of *Streptococcus pneumoniae* (9–13). *Prex1^–/–^* neutrophils also exhibit impaired rolling and intravascular crawling under shear flow conditions, attributed to the reduced activation of the β2-integrins LFA-1 and Mac-1 (14). *In vivo*, Prex1-deficiency compromises immunity against *S. pneumoniae* and impairs neutrophil recruitment during sterile peritonitis, ischemia reperfusion of the kidney, and *S. pneumoniae*-infection of the lung (10, 13–15). Neutrophils deficient in one or more isoforms of Vav show impaired actin polymerisation, adhesion, spreading, migration, phagocytosis, and ROS production (16–20). Neutrophil recruitment is largely unaffected by Vav deficiency (17, 19–21), but the immunity of Vav-deficient mice to *Staphylococcus aureus* and *Pseudomonas aeruginosa* is impaired (19). Vav GEFs also mediate neutrophil-dependent tissue injury during Fc receptor-dependent inflammation of the lung and skin (21). There is cooperation between Prex and Vav Rac-GEFs, as *Prex1^–/–^ Vav1^–/–^* neutrophils show lower cell surface levels of LFA-1 and Mac-1, and have more profound defects in adhesion and migration than cells deficient in the entire Prex family or the entire Vav family (11, 15), and neutrophil recruitment in *Prex1^–/–^ Vav1^–/–^* mice is more strongly impaired during sterile peritonitis and LPS-induced pulmonary inflammation (15). *Dock2^–/–^* neutrophils have reduced actin polarisation, migration speed and phagocytosis, but retain normal β2-integrin mediated adhesion and directional sensing (22–25). Their migration defect is exacerbated by additional Dock5 deficiency (24). Neutrophil recruitment in *Dock2^–/–^* mice is impaired during *Citrobacter rodentium* infection (26) and aseptic peritonitis (25). A human loss-of-function mutation in DOCK2 results in severe immunodeficiency, with impaired neutrophil actin polymerisation, spreading, and ROS production (27). A risk allele associated with decreased DOCK2 expression is seen in patients with severe COVID-19, and inhibition of DOCK2 with the small molecule inhibitor CPYPP increased pneumonia severity in a hamster model of SARS-CoV-2 infection (28). Taken together, loss of adhesion, spreading, polarisation, migration, and anti-pathogen responses of neutrophils are common effects of Rac-GEF deficiencies (4).

T-cell lymphoma invasion and metastasis-inducing protein 1 (Tiam1) is a widely expressed Dbl-type, multi-domain Rac-GEF (29–33). Like most Rac-GEFs, Tiam1 is regulated by autoinhibition, which is relieved upon cell simulation (32, 33). Direct regulators include Ras (34), various protein kinases, and phosphoinositide 3,4,5-trisphosphate, which promotes translocation of Tiam1 from the cytosol to the plasma membrane (32, 33). Studies of Tiam1 function have largely focused on epithelial cells, neurons, and cancer cells. Collectively, they showed that Tiam1 regulates Rac-dependent cytoskeletal dynamics, and thus cell adhesion, polarity, and migration, but also cell growth and survival (30, 32, 33, 35). However, these studies also revealed that Tiam1 function is highly context-dependent, depending on cell type and matrix. For example, Tiam1 can either promote cell migration, as expected for a Rac-GEF, or inhibit migration, the latter generally by fortifying E-cadherin based cell-cell junctions and increasing cell adhesion (32, 36, 37). This translates to complicated roles of Tiam1 in cancer, which can be pro- or anti-oncogenic depending on cancer type and stage (32, 33, 35, 38).

Tiam1 has been characterised in several types of leukocytes, in particular lymphocytes. Tiam1 was originally identified by its ability to render non-invasive T lymphoma cells invasive (29). In T cells, Tiam1 is required for chemotaxis in response to a range of chemoattractants, and mediates crawling on endothelial monolayers under shear flow conditions and transmigration *via* the paracellular rather than transcellular route (39). In adult T cell lymphoma cells, Tiam1 interacts with the immunoglobulin-like cell adhesion molecule Cadm1 and mediates adhesion and spreading (40). In Th17 cells, Tiam1 binds the transcription factor RORγt and is required for the RORγt-dependent expression of interleukin IL-17a (41), and in chronic lymphocytic leukaemia cells, Tiam1 enables the expression of c-Myc (38). *In vivo*, Tiam1 is required for the homing of T cells from the blood stream into various organs (39), and promotes experimental autoimmune encephalomyelitis (41).

We detected Tiam1 in neutrophils during our recent evaluation of the mouse neutrophil proteome (42). Tiam1 has not yet been studied in neutrophils, but its homologue Tiam2 was identified as a target of the transcription factor ATF3 in mouse neutrophils (43). Curiously, knockdown of Tiam2 in neutrophil-like cells derived *in vitro* from mouse hematopoietic stem cells increased actin polymerisation and the size and numbers of focal adhesions, rather than reducing these structures as Rac-GEF deficiency typically would (43). These potentially unique roles of Tiam Rac-GEFs in adhesion and migration compared to other types of Rac-GEFs intrigued us and prompted us to investigate the function of Tiam1 in primary mouse neutrophils. Our study shows that Tiam1 is an important regulator of neutrophil responses, particularly in adherent cells, but exerts this role very differently to other neutrophil Rac-GEFs.

## Materials and methods

### Mice

Tiam1^–/–^ (35), Rac-FRET (Rac-FRET^ki/ki^) (44), *Prex1^–/–^ Vav1^–/–^* (11) and *Dock2^–/–^* (22) mouse strains were described previously. Rac-FRET mice were crossed to *Dock2^–/–^, Prex1^–/–^ Vav1^–/–^* and *Tiam1^–/–^* mice to generate homozygous Rac-FRET *Dock2^–/–^*, Rac-FRET *Prex1^–/–^ Vav1^–/–^* and Rac-FRET *Tiam1^–/–^* strains, respectively (25). RubyLifeact (Lifeact-mRFPruby, RLA) mice (45) were kindly provided by Dr Max Nobis (CRUK Cancer Research Institute, Glasgow, UK), and were bred as heterozygous RLA^tg/+^ animals by crossing with C57Bl6. The Tiam1-RLA strain was generated by crossing *Tiam1^–/–^* and RLA. All mice were on C57Bl6 genetic background. The Biological Support Unit operates as recently described (42). Mice were bred and group-housed (up to 5) under Specific Opportunistic Pathogen Free conditions, with unrestricted access to chow diet and water. They were used in experiments at young-adult age (8-14 weeks). Male mice were used for *in vivo* experiments, and mice of both sexes for *in vitro* experiments, with sex-and age-matching between genotypes. To infect mice with *S. pneumoniae*, they were group-housed in isocages within a biosafety level 2 containment facility. The breeding of animals and experiments were carried out with approval from the local Animal Welfare Ethical Review Body under the British Home Office Animal Scientific Procedures Act 1986.

### Neutrophil isolation

Mature primary neutrophils were purified daily from mouse bone marrow using a Percoll^PLUS^ gradient at 4°C following our previously described method (13). Endotoxin-free reagents were used throughout. The marrow of mouse femurs, tibias, and pelvic bones was flushed out with ice-cold Hank’s Balanced Salt Solution without Ca^2+^ or Mg^2+^ (HBSS^–^, Sigma H6648) supplemented with 15 mM HEPES, pH 7.4 (RT) (Sigma, H3784) and 0.25% fatty acid-free (FAF) BSA (Sigma, A8806) (HBSS^–++^), triturated, and filtered through a 40 μm cell strainer. A 58% isotonic Percoll^PLUS^ solution (GE Healthcare, 17544501) in HBSS^–++^ was added as an underlayer, and the samples were centrifuged at 1620 × g without brake for 30 min at 4°C. After centrifugation, the lower 3 ml were resuspended in 40 ml HBSS^–++^ and centrifuged at 326 × g for 10 min at 4°C. Erythrocytes were lysed using Geye’s solution (130 mM NH_4_Cl, 5 mM KCl, 780 µM Na_2_HPO_4_, 176 µM KH_2_PO_4_, 5.5 mM glucose, 1 mM MgCl_2_, 280 µM MgSO_4_, 1.54 mM CaCl_2_, 13.4 mM NaHCO_3_) for 3 min at RT. 10 volumes of ice-cold HBSS^–++^ were added, and the cells were centrifuged again. The neutrophils were resuspended in ice-cold Dulbecco’s Phosphate Buffered Saline (DPBS) with Ca^2+^ and Mg^2+^ (Sigma, D8662), 1g/l glucose (Sigma, G8769) and 4 mM NaHCO_3_ (Sigma, S8761) (DPBS^++++^) and kept on ice. Aliquots were counted using a haemocytometer and their purity assessed by Kwik-Diff staining (Thermo Scientific Shandon, 9990700) of cytospins. The preparations typically had a purity over 90%. Neutrophils were centrifuged again and resuspended as appropriate for the subsequent assay.

### *S. pneumoniae* infection

Pulmonary infection with *S. pneumoniae* (TIGR4 serotype 4) was carried out essentially as described (13). *S. pneumoniae* of known CFU stored in PBS/20% glycerol at −80°C were thawed, washed twice in DPBS without Ca^2+^ and Mg^2+^ (DPBS^–^, Invitrogen, 14190-094), and resuspended at 4 × 10^7^/ml in ice-cold DPBS^–^. The suspension was kept on ice and used within 1 h. Mice were lightly anaesthetized with 3% isoflurane inhalation, infected intranasally (*i.n.*) with 50 µl *S. pneumoniae* (2 × 10^6^ CFU), or mock-treated with 50 µl DPBS^–^, returned to their home cages, and euthanized by CO_2_ inhalation 6 h later. The femoral artery was severed for confirmation of death, and in some experiments this blood was collected into EDTA-coated microvettes (Sarstedt, 20.1288) for analysis of peripheral neutrophils. Bronchoalveolar lavage (BAL) was performed by three slow injections and aspirations of 1 ml DPBS^–^ through a Venflon cannula (Becton Dickinson, 391452) in the trachea. The BAL fluid was stored on ice. Lungs were perfused with 10 ml DPBS^–^ through the right ventricle, removed using aseptic techniques, and stored on ice. The lungs were minced and homogenised in GentleMACS C tubes (Miltenyi Biotec, 130093237) containing Mouse Lung Dissociation kit enzymes (Miltenyi, 130095927) in DPBS^–^, using a GentleMACS homogenizer (setting 2.1, lung) for 40 s, incubated at 37°C for 30 min, and a second round of homogenisation was performed. The perfused lung homogenate was washed with 10 ml ice-cold DPBS^–^ at 500 x g for 5 min at 4°C. BAL and perfused lung homogenate were serially diluted in ice-cold DPBS^–^, plated onto LB agar containing 5% defibrinated sheep blood (Trafalgar Scientific, DSC025), and CFU were counted. Flow cytometry was performed using a BD Biosciences Fortessa flow cytometer with antibodies, standard beads, and gating as previously described (13). Data were analysed using FlowJo (Tree Star). Neutrophils were identified among single, live CD45^+^ leukocytes, by their characteristic CD11b^hi^, Ly6G^hi^ staining. The volume of BAL fluid recovered was taken into account. To analyse neutrophils in the periphery, blood was collected as described here-above. Red blood cells were lysed in Geye’s solution for 5 min. Leukocytes were washed, incubated in DPBS^++++^, 1% Fc block (BD Biosciences, 553142) for 15 min on ice, washed, stained with BV421-Ly6G (clone 1A8, BioLeged, 127628, 1:500) and AF647-Cd11b (clone M1/70, BD Pharmingen, 557686, 1:1000) antibodies in DPBS^++++^, 1% Fc block for 30 min on ice, washed again, and analysed on a Cytek Aurora flow cytometer.

### Histology

Histological analysis was done essentially as described (13). Mice were infected with *S. pneumoniae*, or mock-infected, and euthanized 6 h later, as described above. Lungs were inflated with 0.5 ml 10% neutral-buffered formalin (Sigma, HT501128), excised, and fixed in formalin. Samples were paraffin embedded, 5 μm sections were prepared, and H&E staining was done by Abbey Veterinary Services (Newton Abbot). Three sections per mouse were imaged on a Nikon Eclipse Ti-E Widefield microscope using the 20× objective and 3 × 3 image-stitching function, generating 2 images of 3 × 3 mm per lung. For image analysis, 6 grids of 40,000 μm^2^ were placed within each image, comprising vascular, interstitial, and epithelial tissue where possible. Neutrophils within the grids were identified by their characteristic nuclear morphology and quantified according to their localisation within 10 μm of the vasculature, bronchiolar or alveolar epithelium, or within the interstitium, using Fiji (ImageJ) (46). 36 grids were quantified per mouse.

To analyse degranulation and NETs, we performed immunohistochemistry (IHC) analysis. Unstained histology slides were deparaffinised in xylene, rehydrated in ethanol, and washed in H_2_O. For antigen retrieval, slides were boiled in 10 mM sodium citrate, pH 6.0, 0.05% Tween-20 for 10 min and allowed to cool to 25°C, and this was repeated with fresh buffer. Samples were washed 3 × 5 min in PBS, permeabilised in PBS, 0.5% Triton-X100 (Sigma, T9284) for 5 min, and washed again. Slides were blocked in 50 mM Tris, 150 mM NaCl, 0.05% Tween 20, pH 7.6 (TBS-T) containing 5% BSA for 1 h at RT. A hydrophobic ring was drawn around the tissue using a PAP pen (Abcam, ab2601), and samples were incubated with myeloperoxidase (MPO, R&D Systems, AF3667, 1:400) and citrullinated histone H3 (CitH3, Abcam, ab5103, 1:1000) antibodies in blocking buffer overnight at 4°C. Samples were washed 3 × 5 min in TBS-T, incubated with anti-goat-IgG-AF647 (ThermoFisher Scientific, A-21447, 1:500) for 30 min at RT, washed again, and incubated with anti-rabbit-IgG-AF488 (ThermoFisher Scientific, A-11034, 1:500) and Hoechst 33342 (ThermoFisher Scientific, 62249, 1:1000) for 30 min at RT. Slides were washed 3 × 5 min in TBS-T, then 2 × 5 min in H_2_O, and mounted using ProLong Gold Antifade (ThermoFisher Scientific, P36934). Samples were imaged as here-above, except using the 4 × 4 stitching function. Image analysis was performed with QuPath, using tissue auto-fluorescence to calculate the total tissue area, and expressing the AF647-positive tissue area as %. In addition, MPO puncta were counted and their relative fluorescence intensities were quantified.

### Thioglycollate-induced peritonitis

Peritonitis experiments were conducted as described (13). Mice were injected *i.p.* with 0.25 ml sterile 3% thioglycollate (TGC, Sigma, T9032) in H_2_O, or mock-treated with H_2_O, and returned to their home cages, with food and water *ad libitum*. 3 h later, the mice were euthanized using CO_2_ asphyxiation, peritoneal lavages performed by two *i.p.* injections, and aspirations of 8 ml DPBS^–^, 5 mM EDTA, and lavage samples pooled and stored on ice. Cells were sedimented at 450 × g for 10 min at 4°C and erythrocytes lysed in Geye’s solution for 3 min at RT. DPBS^++++^ was added, and leukocytes were pelleted and resuspended in DPBS^++++^. Part of each sample was stained with AF647-Cd11b (1:800) and FITC-Gr1 (BD Biosciences, 553126, 1:800) antibodies in DPBS^++++^, 1% Fc block for 20 min on ice in the dark, washed in DPBS^++++^, 5 mM EDTA and resuspended in 500 μl DPBS^++++^, 1 μg/ml DAPI, and 1.25 × 10^5^ Spherotech ACBP-50-10 beads/ml (5.0-5.9 μm). Flow cytometry was done on a BD Biosciences LSRII flow cytometer, and data were analysed in FlowJo. Neutrophils were identified by their Cd11b^hi^, Gr1^hi^ staining. Leukocytes in the remaining sample were counted by haemocytometer and analysed by Kwik-Diff staining of cytospins as an alternative method. The lavage volume recovered was taken into account. Both methods gave similar results.

Where bone marrow transplantation was performed prior to inducing TGC peritonitis, 6-8 week old male C57Bl6 recipient mice were treated with 2 doses of 5 Gray by ^137^Cs γ-irradiation, and their drinking water was supplemented with 2 mg/ml neomycin for two weeks. One day after irradiation, bone marrow cells were purified from 12-week-old male C57Bl6 or Tiam1^–/–^donor mice using aseptic technique. Cells were flushed from bones with DPBS^–^, containing 10% heat-inactivated foetal bovine serum (FBS), filtered through a 40 μm cell strainer, and adjusted to 5 × 10^7^ cells/ml. 100 μl were *i.v.* injected into an irradiated recipient mouse. After 10 weeks, to allow reconstitution of the haemopoietic system, TGC peritonitis experiments were performed as described above.

### ROS production

To measure ROS production between adherent neutrophils, a 96-well luminometer plate was coated with 0.75 µg/ml recombinant mouse ICAM1 (ICAM1/hFc, R&D Systems, 796-IC) in DPBS^–^ overnight at RT, washed 3 times in DPBS^–^, blocked with 2% FAF-BSA in DPBS^–^ for 1 h at RT, and washed 3 × in DPBS^–^ prior to use. Isolated neutrophils at 5 × 10^6^/ml in DPBS^++++^ were primed with 100 ng/ml GM-CSF, 5 ng/ml murine TNFα for 45 min at 37°C. 75 μl cells were added per ICAM1-coated well for 40 min at 37°C, and non-adherent cells were removed by gentle washing with DPBS^++++^. Other primed neutrophils were kept in suspension for 40 min at 37°C, for direct comparison, and 75 μl were dispensed into uncoated wells of the luminometer plate just before the assay. 75 μl prewarmed detection buffer (DPBS^++++^, 16 units/ml horseradish peroxidase (HRP, Sigma, P8375), 120 µM luminol (Sigma, 123072)) was added, and samples were mixed gently and incubated for 3 min at 37°C. 100 μl of prewarmed 7.5 μM fMLP (2.5×, Sigma, F3506) in DPBS^++++^, or DPBS^++++^ control, were added by automatic injection port, and real-time ROS production was recorded at 37°C in a Berthold MicroLumat Plus luminometer (Berthold Technologies). ROS were quantified as the area under the curve (AUC) of the response over 2 min.

ROS production by neutrophils in suspension was measured as described (13). Neutrophils were resuspended and primed with GM-CSF and TNFα as here-above, or were mock-primed in DPBS^++++^. Unprimed neutrophils were maintained on ice and prewarmed to 37°C for 3 min before the assay. fMLP, C5a (Sigma, C5788) and PMA (Sigma, P1585) were prepared as 2.5× stocks in DPBS^++++^. Zymosan A (*S. cerevisiae*, Thermo Fisher, Z2849) were washed twice in DPBS^++++^, opsonised in DPBS^++++^, 50% mouse serum for 1 h at 37°C, or kept unopsonised, and resuspended at 1.125 × 10^7^/ml. Prewarmed detection buffer was added to the cells for 3 min at 37°C as above, before 150 μl were dispensed into a prewarmed, uncoated luminometer plate. 100 μl of prewarmed 2.5× stimuli, or DPBS^++++^, were added by automatic injection port (fMLP, C5a) or manually (PMA, zymosan), and ROS production was recorded in real-time. Final concentrations were 1.5 × 10^6^ neutrophils/ml with 3 μM fMLP, 25 nM C5a, 500 nM PMA, or 4.5 × 10^6^ zymosan A particles/ml. ROS was quantified as the AUC of the response over 2 min for fMLP or C5a, 10 min for PMA, and 90 min for zymosan.

### Degranulation

To measure degranulation in adherent neutrophils, a 12-well plate (Nunc) was coated overnight with 3 µg/ml ICAM1, blocked in 2% FAF and endotoxin-free BSA, DPBS^–^ for 1 h, and washed 3 times in DPBS^–^ before use. An overnight culture of *E. coli* DH5α (New England Biolabs, 2527) was diluted 1:50 in LB and grown to log phase. Bacteria were counted by OD_600_, sedimented, opsonised at 1 × 10^9^/ml in DPBS^++++^, 10% mouse serum for 15 min at 37°C, washed in ice-cold DPBS^++++^, resuspended in DPBS^++++^ at 1 × 10^9^/ml, kept on ice, and prewarmed for 3 min at 37°C before the experiment. Neutrophils at 8 × 10^6^/ml in DPBS^++++^ were allowed to adhere to the ICAM1-coated plate for 30 min at 37°C. In parallel, total lysate controls were prepared by sedimenting cells, resuspending in boiling 1.3× SDS sample buffer, boiling for 10 min, and snap-freezing in liquid nitrogen. After the 30 min incubation, non-adherent cells were carefully aspirated, and 250 µl DPBS^++++^ containing 10 µl (2.5 × 10^7^) opsonised *E. coli* were added to the 3 h timepoint sample. DPBS^++++^ was added to the other samples, and the plate was incubated for 3 h at 37°C. For other timepoints, the DPBS^++++^ was replaced with *E. coli* at 45 and 15 min prior to the end of the incubation, respectively. The supernatant was harvested and cleared by centrifugation for 1 min at 10,000 × g to remove any detached cells. Boiling 4× sample buffer was added to 1.3× final and the sample treated like the total lysates. Samples were western blotted with goat MPO antibody (R&D Systems, AF3667, 1:3000), and the blots were quantified by densitometry using Fiji.

Degranulation of gelatinase (Mmp9) by neutrophils in suspension was measured using in-gel zymography as previously described (13). Neutrophils at 5 × 10^6^/ml in DPBS^++++^ were left unprimed on ice or primed with 50 ng/ml GM-CSF, 20 ng/ml TNFα for 45 min at 37°C. A 96-well plate (Nunc) was blocked with 10% heat-inactivated FBS. 80 μl neutrophils were added to wells containing 20 μl 5× fMLP in DPBS^++++^, or DPBS^++++^alone, and samples were incubated for 30 min at 37°C, 5% CO_2_, followed by centrifugation at 300 × g for 10 min at 4°C. Controls containing cytochalasin B were processed in parallel. The conditioned supernatant was recovered, and 40 μl were mixed with 20 μl 3× non-reducing SDS-PAGE sample buffer (160 mM Tris, pH 6.8, 8% SDS, 50% glycerol, bromophenol blue) at RT. Samples were run on an SDS-PAGE gel containing 0.067% gelatine B. Gels were equilibrated in 2.5% Triton X-100 for 30 min and incubated in 50 mM Tris, pH 7.5, 200 mM NaCl, 5 mM CaCl_2_, 0.02% Triton X-100 overnight at RT, allowing the gelatinase to digest the gelatine. Gels were stained with coomassie, and gelatinase activity was analysed by densitometry using Fiji.

### Release of NETs

NETs formation in response to *S. aureus* (Wood 46) was measured as described (42), except that bacteria were opsonized with 50% mouse serum in Dulbecco’s Modified Eagle Medium with Ca^2+^, Mg^2+^ and 4.5 g/l glucose (Thermo Fisher Scientific, 31053), supplemented with 10 mM Hepes, pH 7.4 (DMEM^++++^), for 30 min at 37°C and resuspended at 2.5 × 10^7^ bacteria/ml in DMEM^++++^. 250 μl neutrophils at 4 × 10^5^ cells/ml in DMEM^++++^, 10% heat-inactivated FBS, were seeded per well of an 8-well μ-slide (ibidi, 80826) and allowed to adhere for 30 min at 37°C, 5% CO_2_. Opsonised *S. aureus* was added at a ratio of 10 bacteria per neutrophil, or cells were mocked-treated with DMEM^++++^, for the indicated periods of time. For each timepoint, the non-cell permeable DNA dye Sytox Green (Thermo Fisher Scientific, S7020, 0.1 mM) and cell permeable Hoechst 33342 (1:500) were added 15 min before the end of the incubation, and samples were live-imaged using a Nikon Eclipse Ti-E widefield system. Images were analysed by Fiji, using phase contrast and Hoechst signals to determine total cell numbers and Sytox Green to enumerate NETs.

### Killing of bacteria *in vitro*

The ability of neutrophils in suspension to kill *S. aureus* was tested essentially as described (13), except that 1 × 10^8^ bacteria were opsonised in 200 μl DPBS^++++^, 50% mouse serum for 30 min at 37˚C, before 800 μl DPBS was added to give 1 × 10^8^ bacteria/ml. Neutrophils at 2.5 × 10^7^/ml in DPBS^++++^ were primed with 50 ng/ml GM-CSF, 20 ng/ml TNFα for 45 min at 37°C. Alternatively, neutrophils were heat-killed for 45 min at 55˚C, then kept on ice, and prewarmed to 37°C for 5 min prior to the assay. 50 μl opsonised bacteria were added to 200 μl primed neutrophils (ratio 1:1), or to heat-killed neutrophils as a control, and incubated at 37°C. 10 μl aliquots were taken after 30 min and 90 min and added to 190 μl ice cold LB, 0.05% saponin, vortexed hard, incubated on ice for 5 min, and vortexed again. Samples containing bacteria but no neutrophils were processed in parallel as an additional control. Serial dilutions in LB were plated onto LB-agar and incubated overnight at 37°C. CFUs of samples with live neutrophils were expressed as % of CFU with heat-killed neutrophils. To measure the ability of adherent neutrophils to kill *S. aureus*, 13 mm coverslips were coated ON with 1 μg/ml ICAM1 in DPBS^++++^. 200 μl neutrophils at 5 × 10^6^/ml in DPBS^++++^ were plated on the coverslips during priming with 50 ng/ml GM-CSF, 20 ng/ml TNFα for 45 min. 50 μl opsonised bacteria at 2 × 10^7^/ml in DPBS^++++^were added (ratio 1:1) and samples incubated at 37°C. Samples were recovered by scraping and processed further as here-above.

### Migration (transwell)

Transwell chemotaxis assays were done essentially as described (11), with 3 μm-pore filters (Millipore, Millicell-PC, PITP01250) in 24-well plates (Costar, 3473). Bone marrow was flushed into HBSS containing Ca^2+^ and Mg^2+^ (HBSS^++^, Sigma, H8264), 0.25% BSA, and 15 mM Hepes, pH 7.5 at 37°C, all endotoxin-free (HBSS^++++^), triturated, strained through 40 μm filters, counted by haemocytometer and resuspended 5 × 10^6^/ml. Cells were primed with 50 ng/ml GM-CSF, 20 ng/ml murine TNFα for 45 min at 37°C, or mock-primed in DPBS^++++^. 400 μl were pipetted onto the transwell filters in wells containing 600 μl prewarmed 1 μM fMLP, 30 ng/ml CXCL1 (KC, Bio-Techne, R&D Systems, 453-KC-010), or 100 ng/ml CXCL1 in HBSS^++++^, or HBSS^++++^ alone, and incubated for 40 min or 90 min at 37°C. Cells remaining in the upper chamber were replaced with 400 μl ice-cold HBSS^–++^, 3 mM EDTA. 60 µl HBSS^–++^, 30 mM EDTA were added to the bottom well, and samples were incubated on iced metal trays for 15 min to detach cells. Cells were collected, centrifuged at 10,000 x g for 30 s, resuspended in ice-cold HBSS^–++^, and stained with FITC-Gr1 (1:800) and AF647-CD11b (1:800) antibodies in HBSS^–++^, 1% Fc block, in parallel to control cells which had not undergone the procedure. Cells were analysed with Spherotech ACBP-50-10 standard beads in an LSR2 flow cytometer, identifying neutrophils by Gr1^hi^/CD11b^hi^ staining.

### Migration (ibidi)

ibidi chamber migration assays were performed essentially as described (25). 6-channel ibidi slides (µ-slide VI 0.4, ibidi 80601) were coated with 3 µg/ml ICAM1 and blocked in BSA as described above, or with 20 μg/ml poly-Arg-Gly-Asp (pRGD, Sigma, F5022), or 1 mg/ml fibrinogen (Sigma, F8630) in DPBS^–^ for 1 h at RT and washed 3 times in HBSS^++++^. The central chamber and wells of the slide were filled with HBSS^++++^. Neutrophils at 2 × 10^7^/ml in HBSS^++++^, 1 g/l glucose were primed with 50 ng/ml GM-CSF, 20 ng/ml TNFα for 45 min at 37°C, or mock-primed. Buffer was removed from the wells, 45 µl cells added into one well, 45 µl liquid removed from the other, and cells were allowed to adhere for 20 min at 37°C. The fMLP gradient was generated by adding 5 μl HBSS^–++^ containing 10 µM fMLP and 5 × 10^6^ carboxyl polystyrene beads (Bangs Laboratories, PC06N, 6.9 μm) into one well, and removing 5 µl buffer from the other. HBSS^–++^ was used for mock-stimulated samples. The location of the beads showed the steepest part of the gradient, where imaging was done. Neutrophils were live-imaged for 20 min at 37°C in an Olympus CellR microscope using the 20× objective, taking frames every 10 s. Cells were tracked manually using the ‘chemotaxis and migration’ plugin of Fiji to quantify speed and directionality.

### Adhesion and migration under shear flow

For imaging under shear-stress, 6-channel ibidi slides (µ-slide VI 0.4, ibidi 80601) were coated with 3 µg/ml ICAM1 and blocked with BSA as described above. Neutrophils at 2 × 10^6^/ml in DPBS^++++^ were stained using CellTracker Deep Red Dye (Invitrogen C34565, 1:2000) for 15 min in the dark, primed with 50 ng/ml GM-CSF, 20 ng/ml TNFα for 45 min at 37°C, and plated into the coated ibidi slide. Cells were allowed to adhere for 15 min at 37°C in a prewarmed environment chamber before non-adherent cells were gently aspirated and the slide was connected to an ibidi flow system. A shear flow of 6 dyn was applied for 10 min during which samples were live-imaged on a Nikon widefield microscope using the 20× objective, acquiring frames every 10 s. Images were analysed using Fiji, with StarDist for cell detection and TrackMate for automated generation of cell tracks. Adhesion was quantified as neutrophils/fields of view (fov) in each frame. Migration was quantified for neutrophils that adhered throughout the experiment. The directionality of migration was expressed as the degree of deviation from a perfect migration path against the shear flow.

### Adhesion and spreading (fixed cells)

Adhesion and spreading were assayed essentially as described (25). Sterile 13 mm glass coverslips (VWR, 631) in a Nunc 24-well plate (Thermo Fisher, 142475) were coated overnight at RT with 20 µg/ml pRGD, 3 µg/ml ICAM1, or 1 mg/ml fibrinogen in DPBS^–^. ICAM1-coated coverslips were blocked with FAF-BSA as described above and all coverslips washed 3 times in DPBS^–^ before use. Neutrophils at 2 × 10^6^/ml in DPBS^++++^ were primed with 50 ng/ml GM-CSF and 20 ng/ml TNFα for 45 min at 37°C, or mock-primed in DPBS^++++^ for 45 min at 37°C, or kept unprimed on ice. Unprimed cells were pre-warmed for 5 min at 37°C before use. 250 µl neutrophils/well were added to 250 µl prewarmed 3 µM fMLP in DPBS^++++^ (2×, for 1.5 µM final), or DPBS^++++^, and incubated for 10 min (pRGD, fibrinogen) or 15 min (ICAM1) at 37°C, 5% CO_2_. Non-adherent cells were aspirated and adherent cells were fixed in 4% paraformaldehyde (PFA), DPBS^++++^ for 15 min at RT. Samples were washed twice in DPBS^–^ and stained with FITC-Gr1 (1:400) antibody, Hoechst 33342 (1:400), and 1% Fc block in DPBS^–^ for 30 min at RT. Coverslips were washed 3 times in DPBS^–^, rinsed in H_2_O, and mounted using Aqua-Poly/Mount (Polysciences, 18806). Samples were imaged on a Nikon Ti-E wide-field fluorescence microscope, using the 3 × 3 image-stitching function at 60× magnification, imaging duplicate coverslips. Images were blinded prior to analysis, a mask was generated for each cell, and all particles larger than 40 μm^2^ were analysed for number, surface area, and circularity using the ‘Set Measurements’ analysis tools of Fiji.

### F-actin and focal complexes (fixed cells)

Sterile 13 mm glass coverslips were coated overnight at RT with 20 µg/ml pRGD or 3 µg/ml ICAM1, and ICAM1-coated coverslips were blocked in FAF-BSA as described above. Neutrophils at 1 × 10^6^/ml were prewarmed for 3 min and allowed to adhere to the coverslips in DPBS^++++^, 0.75 µM fMLP, or in DPBS^++++^ alone, for 15 min at 37°C, 5% CO_2_. Non-adherent cells were aspirated, adherent cells fixed with 4% PFA, DPBS^++++^ for 15 min at RT, and samples washed 3 times in DPBS^–^. Cells were permeabilised for 10 min with 0.1% Triton X-100 in DPBS^–^, washed twice in DPBS^–^, and stained with vinculin-FITC antibody (clone hVIN-1, Sigma, F7053, 1:100) or phalloidin-Atto 655 (Sigma, 18846, 1:100) in DPBS^–^ for 60 min at RT in the dark. Samples were washed twice in DPBS^–^, stained with Hoechst 33342 (1:400) in DPBS^–^ for 5-10 min, washed twice in DPBS^–^ and once in H_2_O, and mounted using Aqua-Poly/Mount. Imaging was done using a Nikon Ti-E wide-field fluorescence microscope with the 100× oil immersion objective. Image analysis was done in a blinded manner to assess neutrophil polarity and F-actin localisation. For TIRF microscopy of focal complexes, neutrophils at 4.5 × 10^6^/ml were primed with 50 ng/ml GM-CSF, 20 ng/ml TNFα in DPBS^++++^ for 45 min at 37°C and seeded onto ICAM1-coated ibidi μ-slide 8-well glass bottom chambers (ibidi, 80827). Neutrophils were stimulated, fixed, and focal complexes stained as here-above, except that samples were kept in DPBS^++++^ during imaging. Neutrophils were imaged on a Nikon Ti2 TIRF microscope using a 60× objective. Images were analysed using Fiji.

### Live-cell imaging of adhesion, morphology, and F-actin dynamics

To image adhesion, morphology, and F-actin dynamics in live neutrophils, ibidi μ-slide 8-well glass bottom chambers were coated with ICAM1 or pRGD, and ICAM1-coated chambers were blocked in FAF-BSA as described above, and prewarmed in the chamber (37°C, 5% CO_2_) of a Nikon Eclipse Ti-E widefield fluorescence microscope. Neutrophils from RLA^tg/+^ and *Tiam1^–/–^* RLA^tg/+^ mice at 4 × 10^6^ cells/ml in DPBS ^++++^ were prewarmed for 3 min at 37°C (pRGD) or primed with 50 ng/ml GM-CSF, 20 ng/ml TNFα for 45 min at 37°C (ICAM1). Neutrophils from one genotype (alternating) were stained with CellTracker as described above and both genotypes were combined in equal quantities, to allow simultaneous live-imaging within one sample. 150 µl mixed RLA^tg/+^ and *Tiam1^–/–^* RLA^tg/+^ neutrophils were added to 150 µl prewarmed 1.5 µM fMLP (2×, for final 0.75 µM) in DPBS^++++^ per well. 4 areas of each slide were live-imaged over 20 min, using the 100× oil immersion objective of a Nikon Eclipse Ti-E widefield system with 400 ms exposure times, acquiring frames every 10 s. Adhesion, spreading, polarisation, and F-actin dynamics were quantified by Fiji, as well as manually measuring actin features using blinded analysis. After analysis, the genotype of each neutrophil was revealed by the CellTracker.

### Morphology of cells in suspension

Neutrophils from RLA^tg/+^ and *Tiam1^–/–^* RLA^tg/+^ mice at 1 × 10^7^/ml in DPBS^++++^ were primed with 50 ng/ml GM-CSF, 20 ng/ml TNFα for 45 min at 37°C, or kept on ice and prewarmed to 37°C for 3 min prior to the assay. 300 μl cells were incubated with 1.5 μM fMLP, or mock-stimulated, for 15 min at 37°C, before being added to 4 ml of ice-cold 4% PFA, DPBS^++++^ and fixed for 15 min at RT. 40 ml PBS was added, and samples were centrifuged at 500 × g for 10 min at 4°C. Samples were washed in PBS and analysed on a Cytek Amnis Imagestream MkII flow cytometer, using a 200 mW 561 nm laser to excite RLA^+^ cells. 10,000 events were acquired per sample. Gating was done on single, RLA^+^ cells in focus on the brightfield. Cells were analysed using IDEAS software for area, circularity (arbitrary scale, high values mean high circularity), aspect ratio (cell width/length), and perimeter.

### Cell surface levels of adhesion molecules

Adhesion molecules on the neutrophil surface were quantified by flow cytometry, essentially as described (13). Bone marrow cells were flushed into ice-cold HBSS^–++^, strained through a 40 μm filter, and enumerated by haemocytometer. Cells were centrifuged at 326 × g for 10 min at 4°C, resuspended in ice-cold DPBS^++++^ at 4 × 10^7^/ml, and either kept on ice to minimise receptor trafficking, or primed with 50 ng/ml GM-CSF, 20 ng/ml TNFα for 45 min at 37°C for maximal receptor upregulation (13, 42, 47). Cells were pelleted and resuspended in an ice-cold cocktail containing FITC-Ly6G (BD Pharmingen, 551460, 1:400), eFluor 450 CD11b (clone M1/70, Invitrogen, 48-0112-82, 1:400) and BV510 CD62L (clone MEL-14, BioLegend, 104441, 1:100) antibodies, 1% Fc block, and one of these PE-conjugated antibodies: PE/Cy7 β1 integrin (CD29, clone HMβ1-1, BioLegend, 102221, 1:100), PE β3 integrin (CD61, clone 2C9.G2, BioLegend, 104307, 1:200), or PE α4 integrin (CD49d, clone R1-2, BioLegend, 103607, 1:200). Another cocktail contained BV510 Ly6G (BioLegend 127633, 1:400) and FITC LFA-1 (clone M17/4, eBioscience, 1:400) antibodies and 1% Fc block. Zombie NIR (BioLegend, 423105, 1:1000) was used as the fixable viability dye. Cells were stained for 20 min on ice, sedimented, resuspended in 1 ml ice-cold HBSS^–++^, 1 mM EDTA, and kept on ice. Flow cytometry was performed in a BD Biosciences Fortessa, and data were analysed by FlowJo. Live single cells were gated, and neutrophils identified by their scatter characteristics and Ly6G^hi^ staining. Adhesion molecule cell surface levels were determined as the mean fluorescence signal of the relevant antibody.

### Integrin affinity and avidity

β2-integrin affinity was measured essentially as described (48). Neutrophils at 1 × 10^7^/ml in DPBS^++++^ were primed with 50 ng/ml GM-CSF, 20 ng/ml TNFα for 45 min at 37°C, followed by 10 min incubation with 2 μg ICAM1, 0.2 μg APC-Fc antibody (Southern Biotech, 9042-11), and 10 μg/ml CD11b antibody (clone M1/70, BioLegend, 101202) on ice. Cells were stimulated with 1.5 μM fMLP or 100 ng/ml CXCL1 in DPBS^++++^, or mock-stimulated in DPBS^++++^, for 3 min at 37°C in a thermomixer at 300 rpm. Control cells were treated with 3 mM Mn^2+^ in DPBS^++++^. Ice-cold 7.4% PFA was added, and cells were fixed for 10 min on ice in the dark. Ice-cold DPBS^–^ was added, and cells were centrifuged for 5 min at 400 x g, 4°C, resuspended in rat anti-mouse Ly6B.2-FITC antibody (clone 7/4, Serotec, MCA771FT, 1:100), and incubated for 10 min on ice in the dark. Cells were washed and resuspended in DPBS^–^. DAPI (Sigma, 10236276001, 1:400) was added and flow cytometry performed on a ZE5 YETI Cell Analyzer (Bio-Rad), using the DAPI signal to gate for live/dead cell populations, FITC for the neutrophil population, and APC to quantify ICAM1 binding.

β2-integrin avidity was measured essentially as previously described (13, 48). Neutrophils at 3 × 10^6^/ml in DPBS^++++^ were primed with 50 ng/ml GM-CSF, 20 ng/ml TNFα for 45 min at 37°C, and incubated with AF594-conjugated activating CD18 antibody (clone M18/2, BioLegend, 101416) (49, 50), during stimulation with 1.5 μM fMLP or 100 ng/ml CXCL1 in DPBS^++++^, or mock-stimulation in DPBS^++++^, for 3 min at 37°C in a thermomixer at 300 rpm. Treatment with 3 mM Mn^2+^ was used as a control. Ice-cold DPBS^–^ was added, and cells were centrifuged at 10,000 x g for 30 s at 4°C, resuspended in 4% PFA and fixed for 10 min on ice, centrifuged at 326 x g for 3 min, resuspended in DPBS^–^, and allowed to settle onto electrostatically charged slides (Superfrost Plus, VWR, 631-0108) for 30 min at RT before mounting in Aqua-Poly/Mount. Cells were imaged using a Nikon confocal A1-R microscope with a 60× 1.4 NA oil objective. CellProfiler and Fiji were used to create cell masks and plot the AF594 signal intensity along the cell periphery. Areas of ≥1.25x median AF594 pixel intensity were considered integrin clusters.

### Rac activity (Pak-CRIB pull down)

Rac activity (GTP loading) in total lysates of neutrophils in suspension was measured by Pak-CRIB pull down assay, as described (44). GST-Pak-CRIB was purified from bacteria and stored in GST-FISH buffer (10% glycerol, 50 mM Tris pH 7.4, 100 mM NaCl, 1% NP-40, 2 mM MgCl_2_, 2 mM DTT, 100 µM PMSF, and 10 µg/ml each of leupeptin, pepstatin A, aprotinin and antipain) at 4°C for no more than a week. 200 μl neutrophils at 1 × 10^7^/ml in DPBS^++++^ were pre-warmed for 3 min at 37°C, and stimulated with various concentrations of fMLP, DPBS^++++^ for 10 s, or mock-stimulated in DPBS^++++^. 1 ml ice-cold GST-FISH buffer, 1.2% NP-40 (final 1% NP-40) was added, and cells were lysed on ice for 2 min with frequent vortexing. Samples were centrifuged at 12,000 × g for 3 min at 2°C, and the supernatant was transferred into fresh precooled tubes. 2% were kept as total lysates. The remaining sample was incubated with GST-Pak-CRIB beads by rotating end-over-end for 15 min on ice. Samples were washed 5 times in GST-FISH buffer, boiling 1.3× SDS-PAGE sample buffer was added, and samples were boiled for 5 min. For the total lysates, boiling 4× SDS-PAGE sample buffer was added (to final 1.3×). Samples were western blotted with Rac1 and Rac2 antibodies. GTP-bound and total Rac were quantified by densitometry using Fiji.

To measure Rac activity in total lysates of adherent neutrophils, 30 mm glass coverslips were coated overnight with 3 μg/μl ICAM1 and blocked with FAF-BSA as described above. 2 ml neutrophils at 1.5 × 10^6^/ml in HBSS^++++^ were plated onto the coverslips in 35 mm tissue culture dishes (Corning) and allowed to adhere for 60 min at 37°C, 5% CO_2_, while priming with 50 ng/ml GM-CSF, 20 ng/ml TNFα. 200 μl 15 μM fMLP in HBSS^++++^ (10×, for 1.5 μM final) were added for 1 min, or cells were mock-stimulated in HBSS^++++^. Dishes were transferred onto an iced metal tray, and the medium was aspirated. 1 ml ice-cold GST-FISH buffer was added, and cells were scraped-off, transferred into pre-cooled Eppendorf tubes, and lysed for 3 min on ice with frequent vortexing. Samples were cleared, incubated with GST-Pak-CRIB, and processed further as described for cells in suspension.

### Live-cell FRET imaging of Rac activity

Live-cell imaging of Rac activity was done essentially as described (25). Ibidi μ-slide 8 well glass bottom slides were coated with ICAM1 or pRGD, and ICAM1 slides were blocked with 2% FAF-BSA as described above. Alternatively, slides were coated with activating CD18 antibody, by incubation with 100 µg/ml poly-D-lysine (Sigma, P6407) and 25 μg/ml protein A (Sigma, P6031) for 1 h at RT, washing, addition of 10 μg/ml activating CD18 antibody (clone M18/2, eBioscience, 14-0181-81) for 1 h at 37˚C, washing again, and blocking in BSA. Slides were washed 3 times in DPBS^++++^ and pre-warmed in the imaging chamber (37°C, 5% CO_2_). For experiments on ICAM1 or pRGD, cells were primed with 50 ng/ml GM-CSF, 20 ng/ml TNFα for 45 min at 37°C, before 150 µl were added to 150 µl prewarmed 1.5 µM fMLP in DPBS^++++^ (2x, for 0.75 µM final) in the ibidi chamber and allowed to adhere for 1 h prior to imaging. For experiments with activating CD18 antibody, neutrophils were kept unprimed and plated without chemoattractant.

Live-cell ratiometric TIRF-FRET microscopy was used to assess the spatiotemporal distribution of Rac activity at the basal cell surface during adhesion and migration. Rac-FRET neutrophils of the various genotypes were directly compared within each experiment, in alternating order. A Nikon Ti2 TIRF microscope with 60× 1.49 NA oil TIRF objective was used with laser excitation at 440 nm. An Andor TuCam emission beam splitter comprising 509 nm dichroic mirror, 480/40 nm (CFP) and 542/27 nm (YFP) band-pass filters (Semrock) was used to direct the fluorescence from the donor channel (CFP) and FRET channel (YFP) to Andor iXon 897 EM-CCD cameras. Channel alignment was done at the start of each experiment using the Fiji plugin bUnwarpJ (51), generating a transformation protocol for images of CPN™ 530 Green standard beads (Stream Bio), which fluoresce in the CFP and YFP channels. This protocol was applied to the whole series of CFP and YFP image pairs. To ensure CFP and YFP images were taken simultaneously, an automated external trigger for the ‘slave’ camera was connected to the ‘master’ camera. TIRF-FRET imaging was done for 120 s, taking frames every 5 s. A mean filter of two pixels was applied and the YFP/CFP ratio image generated as described (44). FRET images were depicted using the Fiji 16-colour table, pseudo-colouring high Rac activity (high FRET ratio) in red and low Rac activity (low FRET ratio) in blue. To quantify overall Rac activity at the basal cell surface, the mean FRET ratio (pixel intensity value) for the whole cell was plotted against time. Alternatively, Rac activity was measured by widefield FRET imaging instead of TIRF-FRET, as previously described (44), to evaluate Rac activity throughout the adhering cell.

Imaging of Rac activity during micropipette chemotaxis assays was done essentially as described (44), except using TIRF-FRET imaging. Briefly, Rac-FRET neutrophils of various genotypes were stimulated with a point source of 0.5 μM fMLP delivered from a femtotip microinjection needle (Eppendorf, 5242957008) and imaged for 2 min by live-cell ratiometric TIRF-FRET imaging, as described above, with a frame interval of 1 s. Line scans were performed through the central longitudinal axis and the pixel with the highest Rac-FRET ratio along this axis determined for each frame. The duration of peak Rac activity localising within 0.8 μm of the leading edge or uropod throughout the 2 min observation period, and the number of oscillations of peak Rac activity between leading edge and uropod, were determined as described (44). In addition, the distance of migration was quantified. Cells that translocated at least 7.8 μm (half their mean body length), during the observation period were considered migrating.

### Isolation and identification of proteins associated with nucleotide-free Rac

GST-Rac1^G15A^ (52) was used to isolate proteins that interact with nucleotide-free Rac. GST-Rac1^G15A^ was purified from bacterial culture, coupled to glutathione sepharose 4B (GE Healthcare, 17-0756-01), and stored in 20 mM Hepes, pH 7.5, 150 mM NaCl, 5 mM MgCl_2_, 30% glycerol, 1 mM DTT, essentially as described (52). 50 mm glass coverslips were coated with 3 μg/μl ICAM1 in 60 mm tissue culture dishes and blocked with 2% FAF-BSA as described above. 4 ml neutrophils at 2 × 10^6^/ml in HBSS^++++^ were allowed to adhere to the coverslips while priming with 50 ng/ml GM-CSF, 20 ng/ml TNFα for 50 min at 37°C, 5% CO_2_, inside a chemical fume hood. 7 mM of the irreversible, cell-permeable protease inhibitor diisopropyl-fluorophosphate (DFP) was added for a further 10 min, before neutrophils were stimulated with 1.5 µM fMLP for 1 min. Dishes were transferred carefully onto an iced metal tray inside the fume hood, the medium aspirated, and samples washed with ice-cold HBSS^++++^. 1 ml ice-cold lysis buffer (20 mM Hepes, pH 7.5, 150 mM NaCl, 5 mM MgCl_2_, 1 mM DTT, 1% Triton X-100, 1:100 protease inhibitor cocktail (Sigma, P8340)) was added, cells were scraped-off, and lysates transferred into precooled Eppendorf tubes. The DFP waste was disposed-of safely, and samples were processed further outside the fume hood. Lysates were incubated for 3 min on ice with frequent vortexing and centrifuged at 12,000 x g for 1 min at 2°C. The supernatant was transferred into fresh precooled tubes and samples were divided. Then 5% was used for total lysates, and the rest divided equally for incubation with either GST-Rac1^G15A^ beads or glutathione sepharose 4B (both prewashed in lysis buffer) by end-over-end rotation for 1 h on ice. Samples were washed 4 times in lysis buffer. For both total lysate and pull down samples, boiling 1.3× SDS-PAGE sample buffer was added, the samples were boiled, and then snap-frozen in liquid nitrogen.

Prior to mass spectrometry, the samples were run ~5 mm into an SDS-PAGE gel, which was stained with Coomassie. The protein-containing region of gel was excised, destained, reduced/alkylated, and trypsin digested as described (53). The resulting peptides were fractionated by high-pH reversed-phase chromatography, using a micro pipette tip packed with Oasis HLB beads. The peptides were loaded in 0.1% ammonium hydroxide and batch eluted with acetonitrile concentrations of 10%, 15%, 20%, and 32%, in 0.1% ammonium hydroxide. The peptide fractions were analysed with an Orbitrap Eclipse mass spectrometer, fitted with a FAIMS Pro interface. Peptides were separated by online reversed-phase nanoLC on an Easy-nLC 1200, with a 90 min gradient of 0-30% acetonitrile (containing 0.1% formic acid), at 300nL/min. The mass spectrometer data acquisition cycle comprised three FAIMS compensation voltages (-40V, -60V, -80V), each of which contained a data-dependent experiment, with one high-resolution MS1 scan followed by 10 MS2 scans, with HCD fragmentation. The mass spectrometric data were searched against the Uniprot mouse proteome using the Mascot search engine within Proteome Discoverer 2.1. Targeted analyses were performed on the same instrument, but without FAIMS and using a 2 h gradient and data-independent analysis. The raw files were processed with DIA-NN software v1.8.1.

Total lysates were western blotted using the following antibodies: 14-3-3 (pan, Cell Signaling Technology, 8312, 1:1000), α-Pix (Cell Signaling Technology, 4573, 1:1000), Git2 (Invitrogen, PA5-78301, 1:1000), Graf1 (Proteintech, 55139-1-AP, 1:1000), Psd4 (Abcam, ab154008, 1:1000), Rap1gds1/SmgGDS (Novus Biologicals, NBP1-87027, 1:1000), Rasa3 (Proteintech, 27835, 1:500), Talin-1 (Cell Signaling Technology, 4021, 1:500), Tiam1 (Bethyl Laboratories, A300-099A, 1:1000) and Tiam2 (54) (1:750).

### Data collection and statistical analysis

Sample size was determined using power calculations to yield 80% power, based on pilot experiments and previously published data. Experiments were conducted at least three times, except where specified. Figure legends contain detailed information on sample size and numbers of independent experiments. Mice were randomly selected for cohorts by the staff of our Biological Support Unit, within specified group size and age parameters. The identity of images was blinded prior to analysis. Excel 2016, GraphPad Prism 9, and R were used for statistical analysis and for making graphs. Data were tested for normal distribution using the Shapiro-Wilk test to determine whether parametric or non-parametric statistical analysis was appropriate. If the variance between groups warranted it, data were log-transformed before statistical analysis, as indicated in the figure legends. Statistical outliers were identified using the ROUT test and excluded from datasets. Other samples were only excluded if there was a known technical problem affecting the analysis. Student’s t-test was used for comparisons between two groups. For *in vivo* experiments with fewer mock-infected mice than infected animals, one-way ANOVA was used, followed by pairwise comparisons between genotypes with Holm-Sidak multiple comparisons corrections, and the resulting multiplicity-adjusted p-values were reported. For all other data, one-way, two-way ANOVA or three-way ANOVA were used as appropriate to test for the effects of interventions, and p-values were reported from the multiplicity-adjusted comparisons. p<0.05 was considered the threshold for statistical significance. Group sizes (n) are listed in the figure legends. P-values denoting significant differences are indicated in the figures in black, and p-values reporting non-significance in grey.

## Results

### Tiam1 is expressed in neutrophils, without affecting neutrophil development, and is required for immunity against pulmonary bacterial infection and for neutrophil recruitment during peritonitis

**Figure 1:**
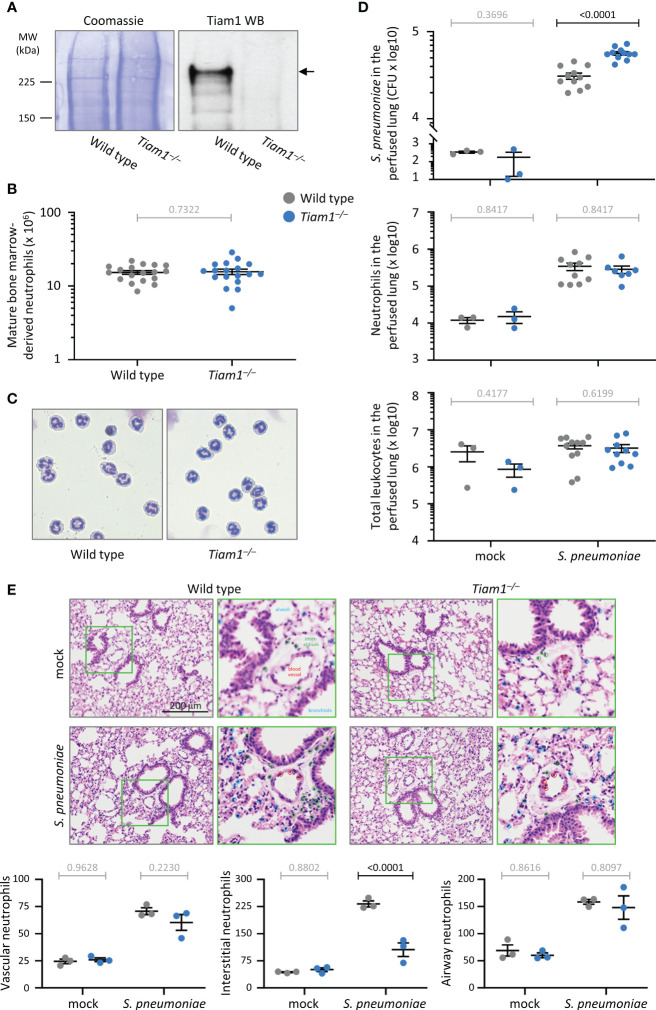
Tiam1 is expressed in neutrophils without affecting neutrophil development, and is required for immunity against pulmonary bacterial infection. **(A)** Total lysates of purified mature bone-marrow derived neutrophils from wild type and *Tiam1^–/–^* mice were western blotted with Tiam1 antibody. Coomassie staining is shown as a loading control. **(B)** Number of mature bone-marrow derived neutrophils isolated from wild type (grey symbols) and *Tiam1^–/–^* (blue symbols) mice. Data are mean ± SEM from 18 independent experiments with 1-2 mice per genotype; each dot represents one experiment. Statistics are paired t-test; grey p-values are not significant. **(C)** Representative Kwick-Diff stained cytospins of purified wild type and *Tiam1^–/–^* neutrophils. **(D)** Infection with *S. pneumoniae*. Wild type and *Tiam1^–/–^* mice were infected *i.n.* with 2 x 10^6^ CFU of *S. pneumoniae*, or were mock-treated and culled 6 h later. Bronchoalveolar lavage (BAL) was performed before lungs were perfused, excised and homogenized. The lung homogenate was cultured for enumeration of *S. pneumoniae* CFU (top panel), or stained for identification of neutrophils (Ly6G^hi^, CD11b^hi^ cells, middle panel) and total leukocytes (single, live CD45^+^ cells, bottom panel) by flow cytometry. Data are mean ± SEM pooled from 3 independent experiments, with 1 mock and 3-4 *S. pneumoniae* treated mice per experiment; each dot represents one mouse. The BAL samples from the same experiments are shown in **Supplementary Figure 1**. Statistics are one-way ANOVA on log-transformed data followed by pairwise comparisons with Holm-Sidak’s multiple comparisons test; p-values in black denote significant differences, p-values in grey are non-significant. **(E)** Histology. Slices of lungs from mice treated as in **(D)** were stained with H&E, and the characteristic nuclear morphology was used to quantify neutrophils according to their localisation within 10 μm of the vasculature (red circles), or bronchiolar and alveolar epithelium (blue circles), or within the interstitium (green circles). Representative images and magnifications of green squares are shown. Data are mean ± SEM of 3 mice per condition, quantifying neutrophils within 6 grids of 40,000 μm^2^ per 3 x 3 mm image, and 6 images per mouse. Statistics are two-way ANOVA with Sidak’s multiple comparison corrections.

To study the role of Tiam1 in neutrophils, we compared primary mouse neutrophils from wild type and *Tiam1^–/–^* mice. Western blots showed that Tiam1 is expressed in wild type neutrophils and deleted in *Tiam1^–/–^* (**Figure 1A**). Neutrophil development was normal in *Tiam1^–/–^* mice, judged by the number and density of neutrophils isolated from the bone marrow, and their characteristic doughnut-shaped nucleus (**Figures 1B, C**).

Neutrophils are the first line of defence against bacterial and fungal infections. To assess whether Tiam1 is important for combatting acute bacterial infection, we infected wild type and *Tiam1^–/–^* mice intranasally with 2 x 10^6^ CFU of *S. pneumoniae* and assessed bacterial titre and neutrophil recruitment into the lung 6 h later. We analysed bronchoalveolar lavages and lung tissue perfused after lavage, to monitor the infection in the airways and the lung tissue. In the Tiam1^–/–^ mice, twice as many bacteria remained in the perfused lung tissue after 6 h than in wild type, which shows that Tiam1 is important for the clearance of bacteria from the lung interstitium and vasculature (**Figure 1D**). *S. pneumoniae*-induced neutrophil recruitment was normal, as were leukocyte numbers overall (**Figure 1D**). In contrast to the perfused tissue, the lavage samples showed normal bacterial titre, neutrophil recruitment, and overall leukocyte number (**Supplementary Figure 1A**), suggesting that Tiam1 is dispensable for clearing the infection from the airways. Neutrophil numbers in the peripheral blood were also normal (**Supplementary Figure 1B**). Therefore, Tiam1 plays a role in innate immunity that depends on the tissue environment.

To assess in more detail if Tiam1 affects the recruitment of neutrophils from the blood vessels into the infected lung, we performed histological analysis, using their characteristic nuclear morphology to identify neutrophils in H&E stained sections. This revealed normal numbers of neutrophils being recruited to the blood vessel wall and into the bronchiolar and alveolar epithelium of *Tiam1^–/–^* mice upon *S. pneumoniae* infection, but reduced numbers in the interstitial space (**Figure 1E**). This suggests that, although overall recruitment was normal, Tiam1 affects the route or speed of neutrophil recruitment during bacterial pneumonia. The reduced neutrophil numbers in the interstitial space likely contribute to the impaired clearance of *S. pneumoniae* in *Tiam1^–/–^* mice.

**Figure 2:**
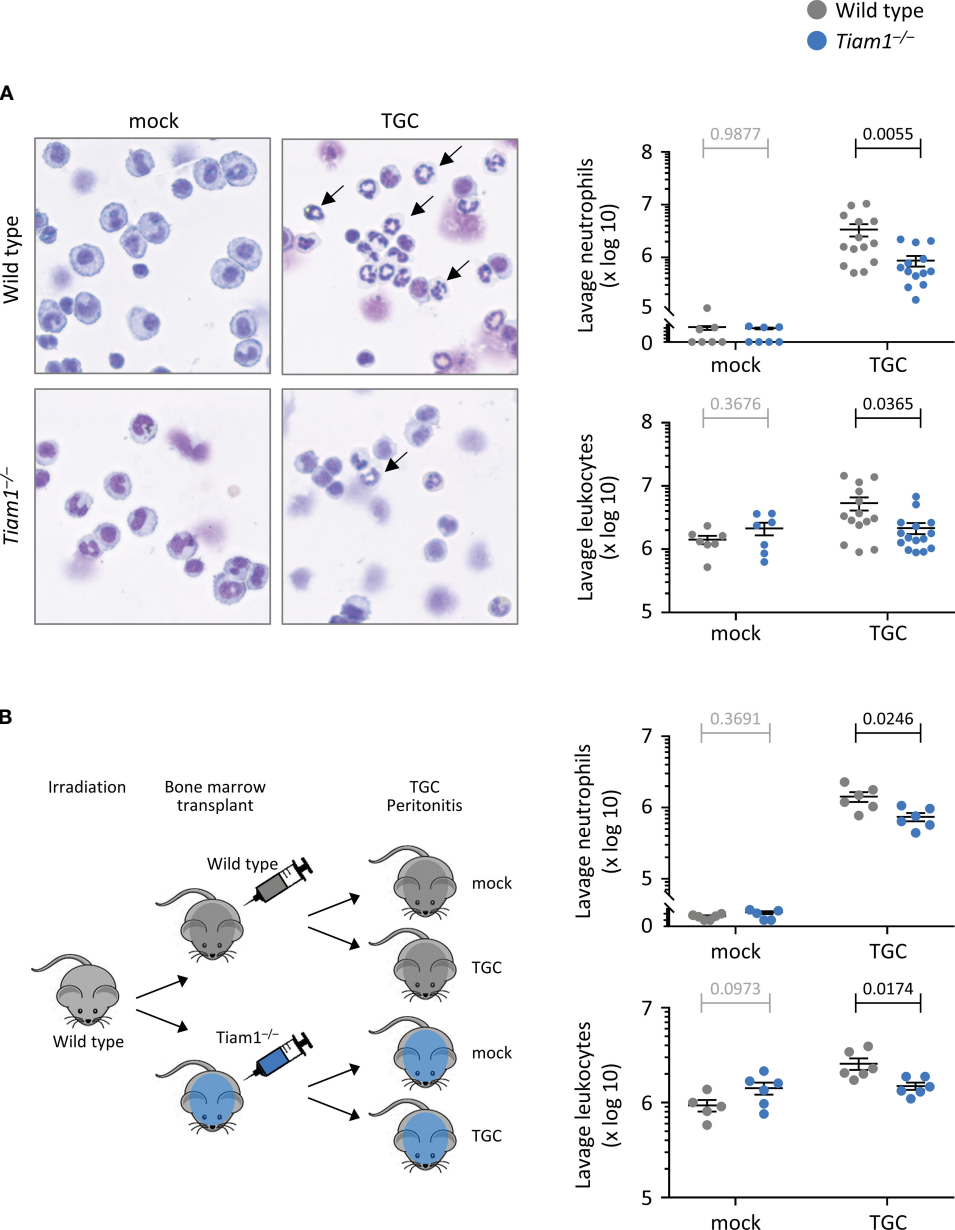
Tiam1 is required for neutrophil recruitment during aseptic peritonitis, via haemopoietic cell-intrinsic mechanisms. **(A)** Wild type (grey symbols) and *Tiam1^–/–^* (blue symbols) mice were treated *i.p.* with 0.25 ml thioglycollate (TGC), or mock-treated, and culled 3 h later. Peritoneal lavages were analysed by Kwick-Diff staining of cytospins, and in parallel by flow cytometry, to identify neutrophils (Gr1^hi^, CD11b^hi^). Representative images show Kwick-Diff stained cytospins; black arrows show examples of neutrophils. Quantifications shown are from cytospin analysis. Data are mean ± SEM pooled from 4 independent experiments, with 1-3 mock and 3-4 TGC treated mice per experiment; each dot represents one mouse. **(B)** Irradiated wild type mice received either wild type or *Tiam1^–/–^* bone marrow cells, and their haematopoietic system was left to recover for 10 weeks before treatment with TGC and analysis as in **(A)**. Data are mean ± SEM pooled from 2 independent experiments, with 1-3 mock- and 3 TGC-treated mice per experiment; each dot represents one mouse. Statistics in **(A, B)** are one-way ANOVA on log-transformed data followed by pairwise comparisons with Holm-Sidak’s multiple comparisons test; p-values in black denote significant differences, p-values in grey are non-significant.

We were interested in studying recruitment also in a different inflammation model, because the requirement for Rac-GEFs in this process is highly organ-specific (1). We investigated neutrophil recruitment in thioglycollate (TGC)-induced sterile peritonitis. *Tiam1^–/–^* mice showed reduced TGC-induced peritoneal neutrophil recruitment, which accounted for decreased overall peritoneal leukocyte numbers (**Figure 2A**). Transplantation of *Tiam1^–/–^* bone marrow into irradiated wild type mice prior to induction of sterile peritonitis confirmed that a *Tiam1^–/–^* haematopoietic system, rather than the tissue environment, was sufficient to cause this reduction in neutrophil recruitment (**Figure 2B**).

Together, these data show that Tiam1 is expressed in neutrophils, is required for antibacterial innate immunity in a manner dependent on the tissue environment, and contributes to neutrophil recruitment during acute inflammation in a manner depending on the tissue environment. The finding that neutrophil numbers were normal overall in the infected *Tiam1^–/–^* lung suggested that impaired neutrophil effector responses may contribute to the reduced immunity.

### Tiam1 is dispensable for neutrophil ROS production, but required for degranulation, NETs release, and killing of bacteria

**Figure 3:**
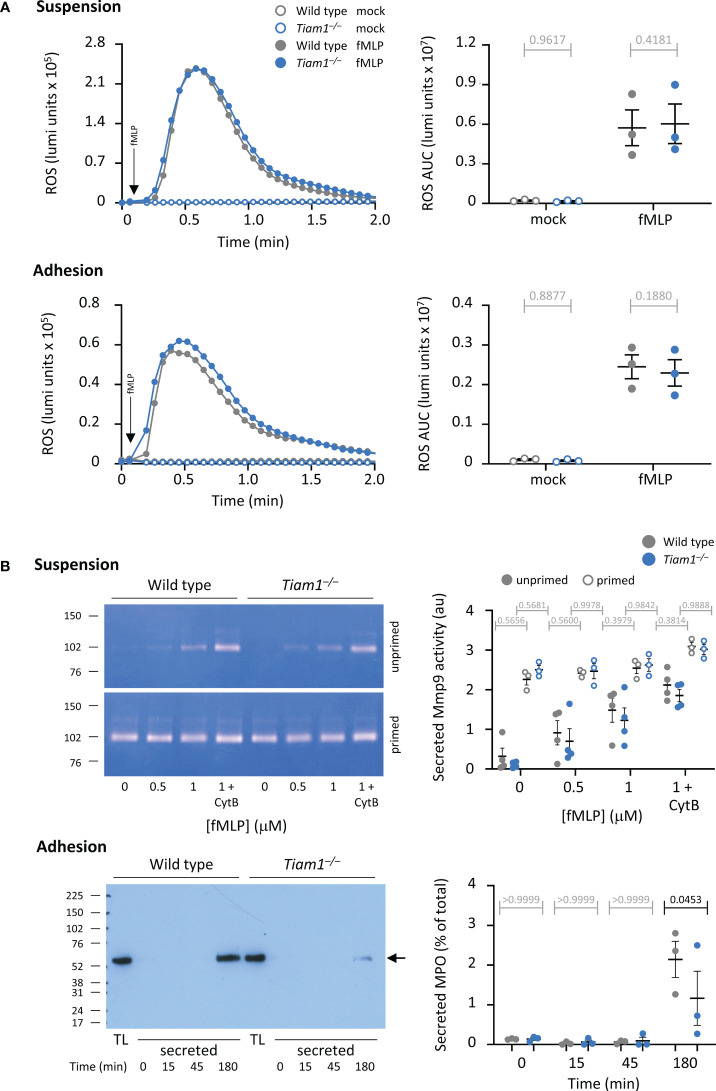
Tiam1 is required for degranulation in adherent neutrophils but dispensable for ROS production. **(A)** ROS production was measured in adherent or suspended wild type (grey symbols) and *Tiam1^–/–^* (blue symbols) neutrophils, as indicated, by luminometer. Neutrophils were primed with 100 ng/ml GM-CSF, 5 ng/ml TNFα for 45 min and then either kept in suspension and added to a luminometer plate just before the assay (top panels), or allowed to adhere to an ICAM1-coated luminometer plate for 40 min at 37°C (bottom panels). HRP and luminol were added, and cells were stimulated with 3 μM fMLP (filled symbols) or mock-stimulated (open symbols). Real-time ROS production was recorded over 2 min. Left: representative curves from one experiment; arrows denote the time of addition of fMLP by automated injection port. Right: ROS production quantified as area under the curve (AUC). Data are mean ± SEM of 3 independent experiments; each dot is the mean of one experiment. **(B)** Degranulation. Top panels: Neutrophils in suspension were kept unprimed (filled symbols) or primed with 50 ng/ml GM-CSF, 20 ng/ml TNFα and for 45 min at 37°C (open symbols) before stimulation with the indicated concentrations of fMLP, and 10 μM cytochalasin B (CytB) where indicated, for 30 min at 37°C, 5% CO_2_. Secreted gelatinase activity was assessed by in-gel zymography and quantified by densitometry. Representative gels are shown. Data are mean ± SEM of raw band intensities from 4 independent experiments. Bottom panels: Neutrophils were allowed to adhere to ICAM1 before incubation with serum-opsonised *E. coli* DH5α for the indicated periods of time. MPO secreted into the medium was quantified by western blotting compared to 1.25% of MPO in the total lysate (TL). Left: representative blot. Right: MPO degranulation as % of total; data are mean ± SEM of 3 independent experiments. Statistics in **(A, B)** are two-way ANOVA with Sidak’s multiple comparison corrections. P-values in black denote significant differences, p-values in grey are non-significant.

A key neutrophil response for killing bacteria is ROS production. We investigated ROS production in neutrophils in suspension in response to a range of stimuli, including the chemoattractants fMLP and C5a, and zymosan yeast particles either in serum-opsonised or unopsonised form. ROS production in response to all stimuli was normal in *Tiam1^–/–^* neutrophils (**Figure 3A**; **Supplementary Figure 2**). Similarly, fMLP-stimulated ROS production in neutrophils adhering to the β2-integrin ligand ICAM1 was also normal (**Figure 3A**), as was receptor-independent ROS production in response to PMA (**Supplementary Figure 2**).

Another neutrophil response important for killing bacteria is degranulation. We measured degranulation both in neutrophils in suspension and in adherent cells. In *Tiam1^–/–^* neutrophils in suspension, the fMLP-stimulated secretion of gelatinase (Mmp9) activity was normal (**Figure 3B**). In contrast, in *Tiam1^–/–^* neutrophils adhering to ICAM1, the *E. coli*-induced degranulation of myeloperoxidase (MPO) was reduced (**Figure 3B**). Hence, Tiam1 is required for degranulation in adherent neutrophils. To test whether this may contribute to the impaired killing of bacteria observed in *Tiam1^–/–^* mice *in vivo*, we measured MPO by IHC in lung histology sections. However, no significant changes in MPO signal were observed under these conditions, although there was a trend to increased MPO puncta in *S. pneumoniae*-infected *Tiam1^–/–^* mice (**Supplementary Figure 3**).

**Figure 4:**
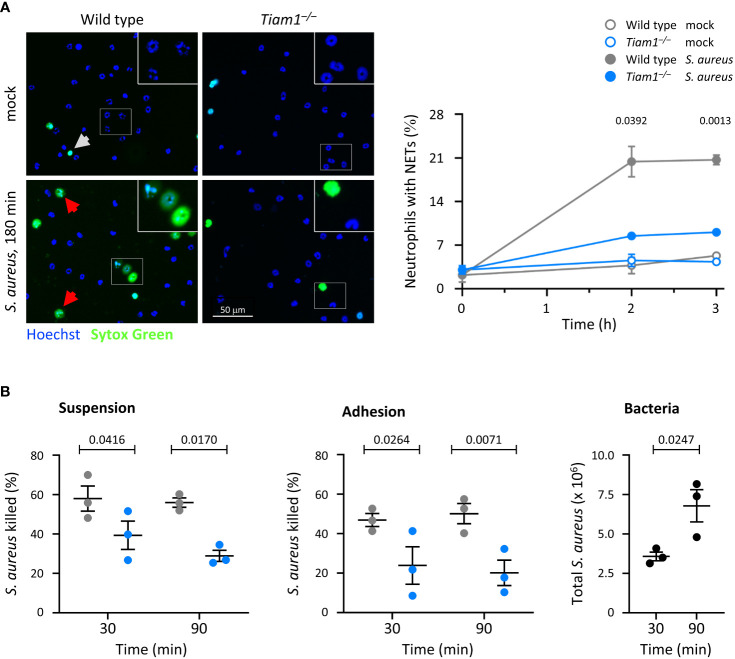
Tiam1 is required for NETs release and killing of bacteria by neutrophils. **(A)** NETs. Wild type (grey symbols) and *Tiam1^–/–^* (blue symbols) neutrophils were allowed to adhere to ibidi μ-slides for 30 min before the addition of serum-opsonised *S. aureus* (ratio 10 bacteria per neutrophil), or mock-treatment. 15 min before a timepoint, Sytox Green and Hoechst 33342 were added, and cells were live-imaged. Representative images from one experiment are shown. White squares denote the magnified areas. White arrows highlight dead cells, red arrows NETs. Quantification by image analysis; data are mean ± SEM of 3 independent experiments. Statistics are two-way ANOVA with Sidak’s multiple comparison corrections on log-transformed raw data. **(B)** Killing of bacteria. Wild type and *Tiam1^–/–^* neutrophils were primed with 50 ng/ml GM-CSF, 20 ng/ml TNFα for 45 min either in suspension or while adhering to ICAM1-coated coverslips, as indicated. Serum-opsonised *S. aureus* (ratio 1 bacteria per neutrophil) were added for 30 or 90 min, before samples were permeabilised with saponin, plated on LB-agar, and CFU of surviving bacteria enumerated. Samples with heat-killed neutrophils were processed in parallel to determine the efficacy of killing. Samples containing bacteria but no neutrophils (black symbols) were used as an additional control (right-hand panel). Data are mean ± SEM of 3 independent experiments. Statistics are two-way ANOVA with Sidak’s multiple comparisons corrections.

Neutrophils release NETs to kill bacteria. We measured NET release in response to *S. aureus*, which was impaired in *Tiam1^–/–^* neutrophils (**Figure 4A**). This impairment likely contributes to the reduced clearance of bacteria *in vivo*, although this could not be verified, as CitH3 signal was too rare to be evaluated in a meaningful way by IHC in lung histology sections of *S. pneumoniae*-infected mice under the conditions tested (data not shown).

To test directly if Tiam1 is required for the killing of bacteria, we incubated neutrophils in suspension or adhering to ICAM1 with *S. aureus* for 30 or 90 min. The killing of *S. aureus* was reduced in *Tiam1^–/–^* neutrophils under all conditions tested (**Figure 4B**). Hence, Tiam1 is required for the killing of bacteria both in neutrophils in suspension and in adherent neutrophils. This defect, together with the altered distribution of neutrophils in the lung, can explain the reduced immunity of *Tiam1^–/–^* mice.

### Tiam1 is required for β2-integrin dependent neutrophil chemotaxis but limits random migration under shear stress

**Figure 5:**
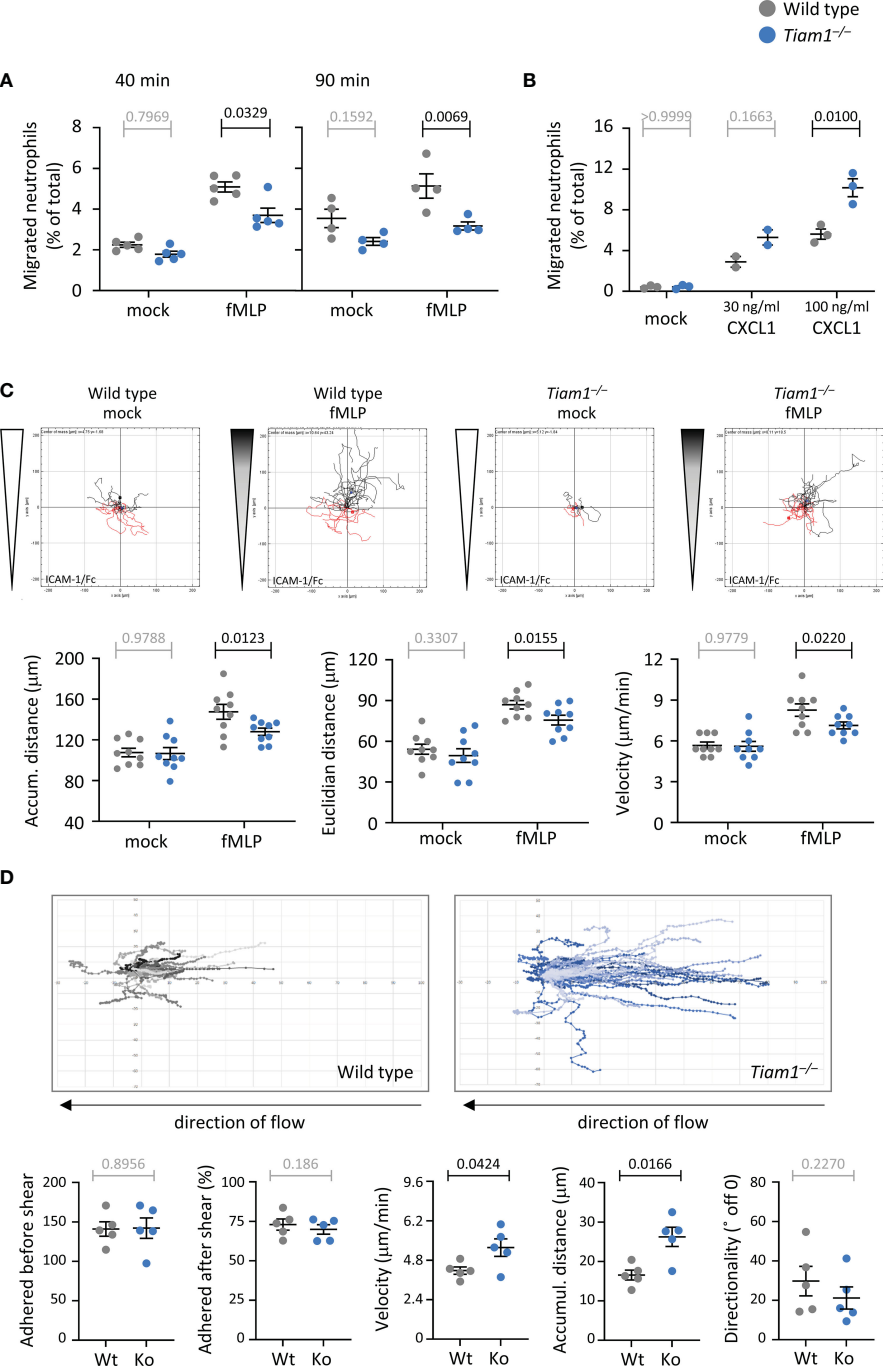
Tiam1 is required for neutrophil chemotaxis but limits migration under shear stress. **(A)** Transwell chemotaxis with fMLP. Wild type (grey symbols) and *Tiam1^–/–^* (blue symbols) bone marrow cells were primed with 50 ng/ml GM-CSF and 20 ng/ml TNFα for 45 min at 37˚C before seeding into the upper well of a transwell chemotaxis chamber (3 μm pores) with either 1 μM fMLP or buffer (mock) in the lower chamber and incubation for 40 or 90 min, as indicated. Neutrophils recovered from the lower chamber were identified by flow cytometry. Data are mean ± SEM of 4-5 independent experiments; each dots is the mean of one experiment. Statistics are three-way ANOVA with Sidak’s multiple comparisons test. **(B)** Transwell chemotaxis with CXCL1. Wild type and *Tiam1^–/–^* cells were treated as in **(A)** except with 30 ng/ml or 100 ng/ml CXCL1 as the chemoattractant for 40 min. Data are mean ± SEM of 3 independent experiments (2 for 30 ng/ml). Statistics are two-way ANOVA with Sidak’s multiple comparisons test. **(C)** Chemotaxis on ICAM1. Purified wild type and *Tiam1^–/–^* neutrophils were primed as in **(A)**, plated into an ICAM1-coated ibidi chamber (µ-slide VI 0.4), and their migration in a chemoattractant gradient with 10 µM fMLP as the highest concentration (shaded wedges, fMLP), or in buffer only (white wedges, mock), was imaged for 20 min. Cells were tracked in the steepest area of the gradient. Tracks from one representative experiment are shown. Data are mean ± SEM of 9 independent experiments; each dot is the mean of one experiment. **(D)** Adhesion and migration on ICAM1 under shear-stress. Wild type and *Tiam1^–/–^* neutrophils were primed as in **(A)** and plated into ICAM1-coated 6-channel ibidi slides. Cells were left to adhere for 15 min at 37°C before shear flow of 6 dyn was applied, and samples were live-imaged by widefield microscopy for 10 min, with image acquisition every 10 s. Cell tracks were generated and analysed for the indicated behaviours using Fiji. Representative tracks from one experiment are shown. Data are mean ± SEM of 5 independent experiments, with 6 samples per genotype in each experiment; each dot is the mean of one experiment. Statistics in **(B, C)** are two-way ANOVA with Sidak’s multiple comparisons test. **(A–C)** P-values in black denote significant differences, p-values in grey are non-significant.

We investigated neutrophil migration, initially using transwell chemotaxis assays where neutrophils migrate through 3 μm pore filters. fMLP-stimulated chemotaxis was impaired in *Tiam1^–/–^* neutrophils at both timepoints investigated, 40 min and 90 min, whereas random neutrophil migration in the absence of chemoattractant was normal (**Figure 5A**). Hence, Tiam1 is required for fMLP-induced neutrophil chemotaxis. To test if a similar defect can be observed in response to another chemoattractant, we did transwell assays with CXCL1 (KC), the mouse equivalent of human IL8. Interestingly, CXCL1-induced chemotaxis was increased in *Tiam1^–/–^* neutrophils, rather than decreased (**Figure 5B**). This shows that Tiam1 plays an interesting role in promoting or limiting neutrophil chemotaxis, depending on the chemoattractant.

To study migration in more detail, we tracked the paths of migrating neutrophils in an ibidi chemotaxis chamber. Neutrophils adhere and migrate in an integrin-dependent manner. The main integrin types expressed in neutrophils are β1 and β2 (55), although we also detected β3, β5, and β7 in our recent proteomic analysis (42). Hence, we coated the ibidi chamber with various integrin ligands: ICAM1, the archetypical β2 integrin-ligand; pRGD, a promiscuous ligand for β1 and other integrins; or fibrinogen, which predominantly binds β3 integrins (56–58). In *Tiam1^–/–^* neutrophils plated on ICAM1, fMLP-stimulated chemotaxis was reduced, whereas random migration in the absence of chemoattractant was unchanged (**Figure 5C**), as previously seen in the transwell assay. Chemotaxis was reduced both in terms of accumulated distance and Euclidian distance (as the crow flies). The impairment resulted from decreased velocity of migration, whereas directional sensing and the propensity to migrate were normal (**Figure 5C**; **Supplementary Figure 4**). Migration on pRGD was also impaired, but in a different manner. The fMLP-stimulated chemotaxis of *Tiam1^–/–^* neutrophils on pRGD was normal, whereas random migration in the absence of chemoattractant was increased, due to increased velocity (**Supplementary Figure 5**). On fibrinogen, both random migration and chemotaxis were normal (**Supplementary Figure 6**). Hence, Tiam1 plays complex roles in neutrophil migration that vary depending on the chemoattractant and the integrin ligand through which the neutrophils adhere. Judging by cell behaviours on the various ligands, we conclude that Tiam1 is required for β2-integrin dependent neutrophil chemotaxis and may limit β1-dependent random migration, but is likely dispensable for β3-dependent migration.

During recruitment *in vivo*, neutrophils become adherent to the endothelial wall of venules under shear stress, and then migrate against the direction of blood flow until they find a site suitable for transmigration (59). To mimic this situation, we applied a shear flow of 6 dyn/cm^2^ to GM-CSF/TNFα-primed neutrophils plated on ICAM1 (60). *Tiam1^–/–^* neutrophils adhered normally both before and after the application of flow, and both genotypes migrated against the direction of flow, but the *Tiam1^–/–^* cells migrated much further, due to increased velocity rather than altered directionality (**Figure 5D**). Hence, Tiam1 limits random neutrophil migration on ICAM1 under shear flow, as it does on pRGD under static conditions. Together, the complex, context-dependent roles of Tiam1 in neutrophil migration suggested that Tiam1 may play similarly complex roles in controlling the cytoskeletal dynamics that underlie adhesion and migration.

### Tiam1 controls neutrophil adhesion, polarisation, F-actin dynamics, and focal complexes

**Figure 6:**
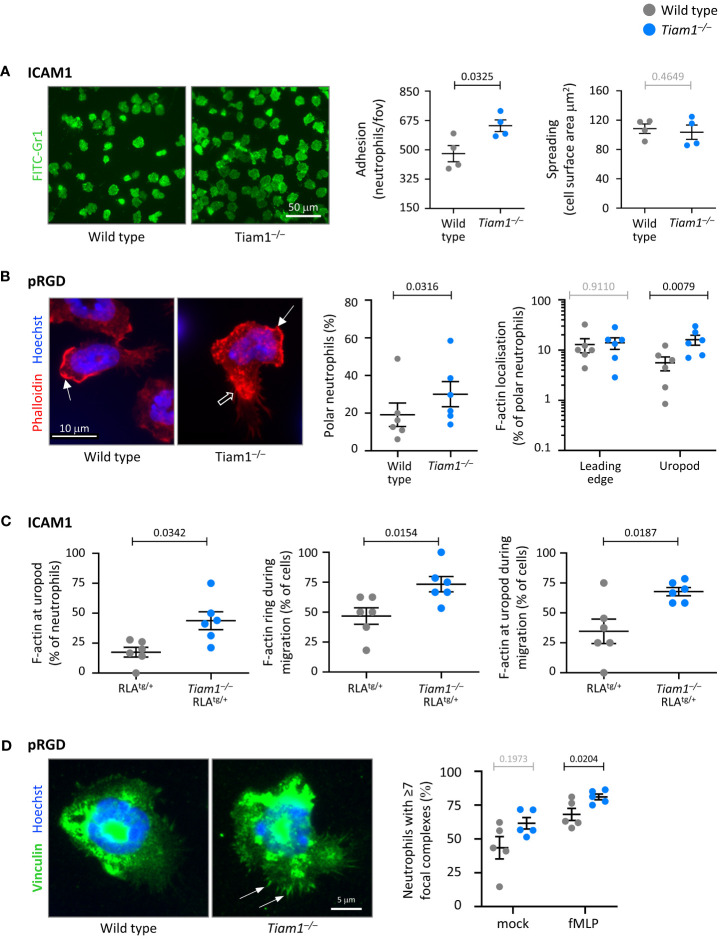
Tiam1 limits neutrophil adhesion, polarity and focal adhesions, and determines F-actin dynamics. **(A)** Adhesion and spreading. Wild type (grey symbols) and *Tiam1^–/–^* (blue symbols) neutrophils were prewarmed, plated onto ICAM1-coated coverslips for 10 min at 37˚C, fixed, stained with FITC-Gr1 antibody and imaged by widefield fluorescence microscopy. Representative images from one experiment are shown. Cell masks were generated. Adhesion was quantified as the number of neutrophils per fov; spreading as the surface area of each cell mask. Data are mean ± SEM of 4 independent experiments, with 27 fov per coverslip and duplicate coverslips per condition assessed in each experiment; each dot is the mean of one experiment. Statistics are two-way ANOVA with Sidak’s multiple comparisons test. **(B)** Cell and F-actin polarity. Wild type and *Tiam1^–/–^* neutrophils were allowed to adhere to pRGD-coated coverslips for 15 min at 37°C, fixed, permeabilised, and stained with phalloidin-Atto 655 and Hoechst 33342. Cell morphology and F-actin localisation were analysed using widefield fluorescence microscopy, with images blinded prior to analysis. Representative images from one experiment are shown. Closed arrows denote F-actin at the leading edge, open arrows F-actin at the uropod. Data are mean ± SEM of 6 independent experiments, with 35-96 cells per genotype assessed in each experiment; each dot is the mean of one experiment. Statistics for cell polarity are paired t-test, for F-actin distribution two-way ANOVA with Sidak’s multiple comparisons test. **(C)** F-actin dynamics. RubyLifeact (RLA^tg/+^) and *Tiam1^–/–^* RLA^tg/+^ neutrophils were primed with 50 ng/ml GM-CSF, 20 ng/ml TNFα for 45 min at 37˚C. RLA^tg/+^ neutrophils were stained with CellTracker, mixed with unstained *Tiam1^–/–^* RLA^tg/+^ neutrophils, plated onto ICAM1 with 0.75 µM fMLP, and live-imaged by widefield fluorescence microscopy for 20 min from the moment they started to adhere. See also **Supplementary Movie 1**. Data are mean ± SEM of 6 independent experiments, with movies of 90 RLA^tg/+^ and 131 *Tiam1^–/–^* RLA^tg/+^ neutrophils analysed; each dot is the mean of one experiment. Statistics are paired t-test. **(D)** Focal complexes. Wild type and *Tiam1^–/–^* neutrophils were allowed to adhere to pRGD for 15 min at 37°C in the presence or absence of 0.75 µM fMLP. Cells were fixed, permeabilised, stained with vinculin antibody and Hoechst 33342, imaged by widefield fluorescence microscopy, and images blinded prior to analysis. Representative images are from one experiment in the presence of fMLP; arrows denote focal complexes. Data mean ± SEM of 5 independent experiments, with 37-80 cells analysed per condition; each dot is the mean of one experiment. Statistics are two-way ANOVA with Sidak’s multiple comparisons test. **(A–D)** P-values in black denote significant differences, p-values in grey are non-significant.

We investigated the roles of Tiam1 in neutrophil adhesion, polarity, and cytoskeletal properties, both on ICAM1 and pRGD. Adhesion of *Tiam1^–/–^* neutrophils to ICAM1 was increased, whereas adhesion to pRGD was normal, and spreading was normal on both surfaces (**Figure 6A**; **Supplementary Figure 7A**). The increased adhesion on ICAM1 was only seen in unprimed *Tiam1^–/–^* neutrophils, whereas GM-CSF/TNFα-primed cells adhered normally (**Figures 5D**, **6A**, and data not shown).

To investigate neutrophil polarisation and F-actin distribution, we allowed wild type and *Tiam1^–/–^* neutrophils to adhere to ICAM1 or pRGD, fixed them, stained their F-actin, and analysed them by fluorescence microscopy. On pRGD, more *Tiam1^–/–^* neutrophils polarised more than wild type, whereas on ICAM1 it was the inverse (**Figure 6B**; **Supplementary Figure 7B**). Consistent with the altered neutrophil polarity, F-actin distribution was also affected. Usually, F-actin is mostly localised at the leading edge of polar neutrophils. However, in *Tiam1^–/–^* neutrophils, there was increased localisation of F-actin also at the uropod (**Figure 6B**; **Supplementary Figure 7C**), which may contribute to the impaired migration.

To investigate F-actin dynamics in live cells, we crossed the *Tiam1^–/–^* mouse to a reporter mouse with fluorescently-labelled actin, RubyLifeact (RLA) (45), and compared wild type RLA^tg/+^ with *Tiam1^–/–^* RLA^tg/+^ neutrophils. We stained cells from one genotype with CellTracker, mixed cells from both genotypes within the same sample, plated them on ICAM1 or pRGD, and live-imaged them in the presence of fMLP. Neutrophils of both genotypes polarised and underwent chemokinesis, but the *Tiam1^–/–^* cells had altered F-actin dynamics and more F-actin at their uropod on both surfaces. On ICAM1, *Tiam1^–/–^* RLA^tg/+^ neutrophils maintained their cortical F-actin ring and F-actin accumulation at the uropod even during migration (**Figure 6C**; **Supplementary Figure 7C, Movie 1**). On pRGD, the cortical F-actin ring was more pronounced, maintained for longer, and reformed during chemokinesis (**Supplementary Figures 7C, D, Movie 2**). Hence, Tiam1 regulates neutrophil polarisation and F-actin dynamics during adhesion and chemokinesis.

To test if Tiam1 also affects the morphology of neutrophils in suspension, we analysed RLA^tg/+^ and *Tiam1^–/–^* RLA^tg/+^ neutrophils by imagestream flow cytometry. fMLP stimulation and/or priming of neutrophils caused the RLA^tg/+^ cells to spread, elongate, and form protrusions. In contrast, *Tiam1^–/–^* RLA^tg/+^ neutrophils showed these morphologies constitutively, and fMLP stimulation or priming did not further enhance this (**Supplementary Figure 8**). Hence, as during adhesion, Tiam1 also controls the morphology of neutrophils in suspension, where it maintains their rounded basal state.

Focal adhesions connect extracellular matrix-bound integrins to the actin cytoskeleton. Neutrophils do not make the long-lived focal adhesions typical of cultured cells but instead form smaller, short-lived adhesive structures, which are sometimes termed focal complexes (43, 61). We investigated focal complexes in wild type and *Tiam1^–/–^* neutrophils adhering to pRGD or ICAM1, by staining with the marker vinculin. Widefield microscopy showed that *Tiam1^–/–^* neutrophils on pRGD had more focal complexes than wild type upon stimulation with fMLP (**Figure 6D**). TIRF microscopy showed that *Tiam1^–/–^* neutrophils on ICAM1 had the same number of focal complexes as wild type, but these focal complexes were smaller under basal conditions (**Supplementary Figure 7E**). Hence, Tiam1 controls focal complex number or size, depending on the integrin ligand through which the cells adhere. As focal complexes reflect the number or activity of integrins, this suggested that Tiam1 may regulate the cell surface levels of integrins, or the affinity and avidity of integrins.

### Tiam1 controls neutrophil β2 integrin affinity and avidity

**Figure 7:**
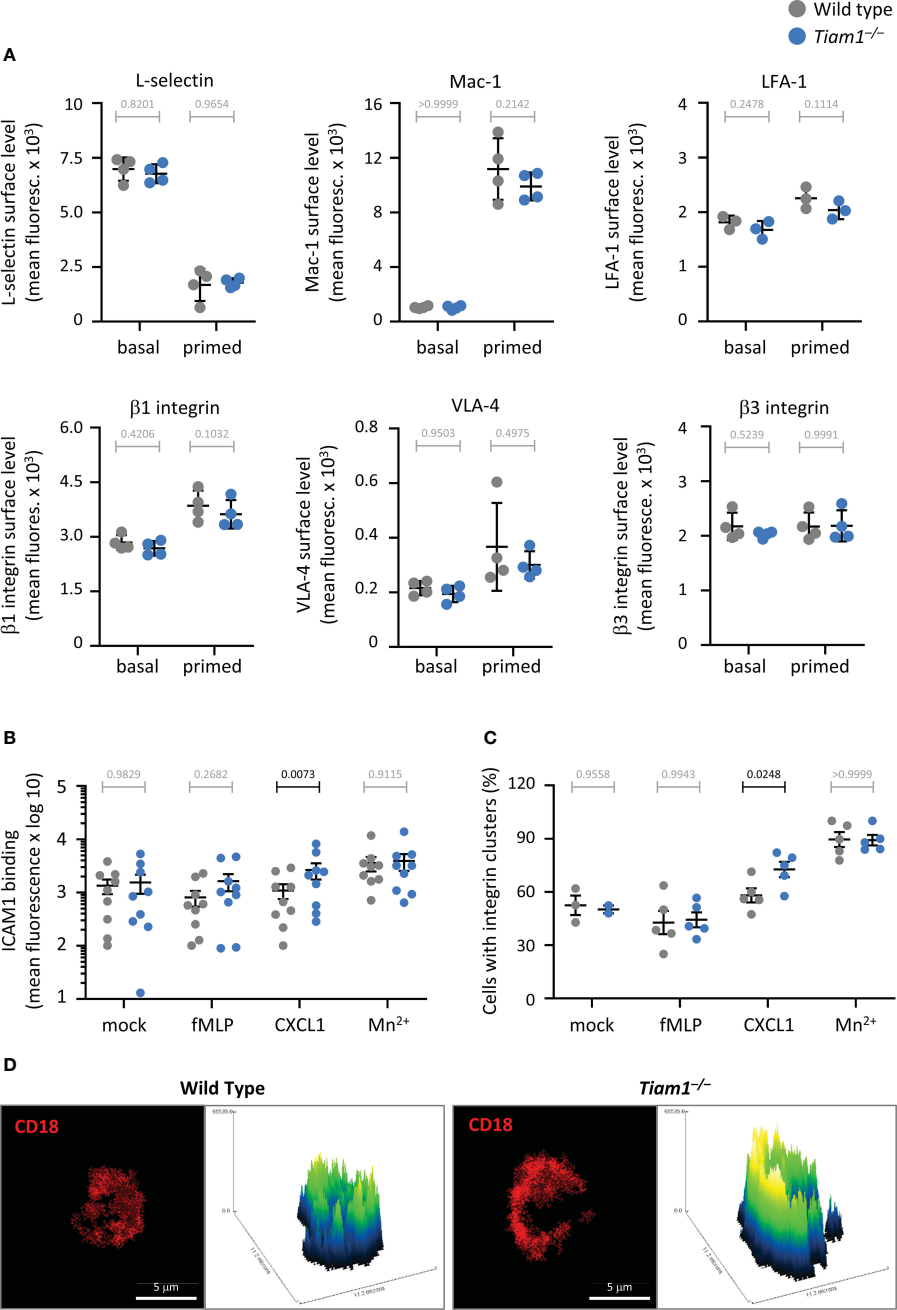
Tiam1 limits CXCL1-stimulated β2-integrin affinity and avidity. **(A)** Cell surface levels of adhesion molecules. Wild type (grey symbols) and *Tiam1^–/–^* (blue symbols) neutrophils were kept basal or primed with 50 ng/ml GM-CSF, 20 ng/ml TNFα for 45 min at 37˚C prior to staining and analysis by flow cytometry for the cell surface levels of the indicated adhesion molecules. Data are mean fluorescence intensity ± SEM of 4 independent experiments; statistics are two-way ANOVA with Tukey’s multiple comparisons test. **(B)** ICAM1 binding. Wild type and *Tiam1^–/–^* neutrophils were primed as in **(A)** and stimulated with 1.5 μM fMLP or 100 ng/ml CXCL1 (KC), or mock-stimulated, for 3 min at 37°C in the presence of ICAM1. 3 mM Mn^2+^ was used as a positive control. Cells were fixed, stained with Ly6B.2-FITC antibody and analysed by flow cytometry for ICAM1 binding. Data are mean ± SEM of 9 independent experiments; each dot is the mean of one experiment. Statistics are two-way ANOVA on log-transformed data with Sidak’s multiple comparisons test. **(C, D)** β2-integrin avidity. Wild type and *Tiam1^–/–^* neutrophils were primed as in **(B)** and stained with CD18-Alexa594 antibody before stimulation as in **(B)**. Cells were fixed, allowed to settle onto electrostatically charged slides, and imaged by confocal microscopy. **(C)** Quantification of integrin clusters. Data are mean ± SEM of 3-5 independent experiments, with 30-40 cells per condition analysed for each experiment; each dot is the mean of one experiment. Statistics are two-way ANOVA with Sidak’s multiple comparisons test. **(D)** Representative images from one experiment of cells stimulated with CXCL1, showing 3-D plots of β2 integrin clusters. **(A–C)** P-values in black denote significant differences, p-values in grey are non-significant.

To test for cell surface levels of adhesion molecules, we performed flow cytometry experiments in basal and primed neutrophils. Neutrophils reacted to priming by strongly upregulating the β2 integrin Mac-1 (p=0.0008 in wild type) onto their surface, and shedding L-selectin (p=0.0008), whereas β2 integrin LFA-1 increased only slightly (p=0.0290) (**Figure 7A**), as expected (13, 42, 47). The β-chain common to β1 integrins was also upregulated slightly upon priming (p=0.0106), whereas the α-chain of the β1 integrin VLA-4 (α4β1) and the β-chain common to β3 integrins remained unchanged. Importantly, the cell surface levels of all adhesion molecules tested were the same between wild type and *Tiam1^–/–^* neutrophils (**Figure 7A**). Therefore, Tiam1 does not affect the cell surface levels of major neutrophil adhesion molecules.

β2 integrins must be activated from their bent, closed conformation to their extended, open conformation in order to bind ICAM1, and this activation occurs in response to chemoattractant signalling through GPCRs, or can be artificially induced by exposure to Mn^2+^ (62). We tested the ability of neutrophils to bind ICAM1 by flow cytometry. ICAM1 binding increased upon Mn^2+^ treatment, as expected, and to the same extent in both wild type and *Tiam1^–/–^* neutrophils, suggesting that the overall ICAM1 binding capacity of *Tiam1^–/–^* neutrophils is normal, in line with the normal β2 integrin cell surface levels. Stimulation with fMLP had no apparent effect under the primed conditions tested, but CXCL1 induced an increase in ICAM1 binding in *Tiam1^–/–^* neutrophils, although not in wild type (**Figure 7B**).

Active β2 integrins have higher avidity, meaning they cluster within the membrane to facilitate adhesion. We tested β2 integrin avidity by microscopy. Similar to ICAM1 binding, Mn^2+^ treatment increased clustering equally in wild type and *Tiam1^–/–^* neutrophils. Stimulation with fMLP had again no effect, whereas CXCL1 increased β2 integrin clusters and the size of these clusters in *Tiam1^–/–^* neutrophils but not in wild type (**Figures 7C, D**). Together, these data show that Tiam1 limits CXCL1-stimulated β2 integrin affinity and avidity in neutrophils, despite normal β2 integrin cell surface levels. This may explain how Tiam1 limits CXCL1-dependent chemotaxis.

### Tiam1 limits the activation of Rac during β2-integrin dependent neutrophil adhesion

We were intrigued by the increased adhesion and migration under shear flow of *Tiam1^–/–^* neutrophils on ICAM1, by their increased polarity and random migration on pRGD, and by the increased chemotaxis towards CXCL1, because deficiencies in Rac and Rac-GEFs would usually reduce these responses. To test whether these increased cell responses may be a reflection of altered Rac activity, we tested the fMLP-stimulated activation of Rac in wild type and *Tiam1^–/–^* neutrophils.

**Figure 8:**
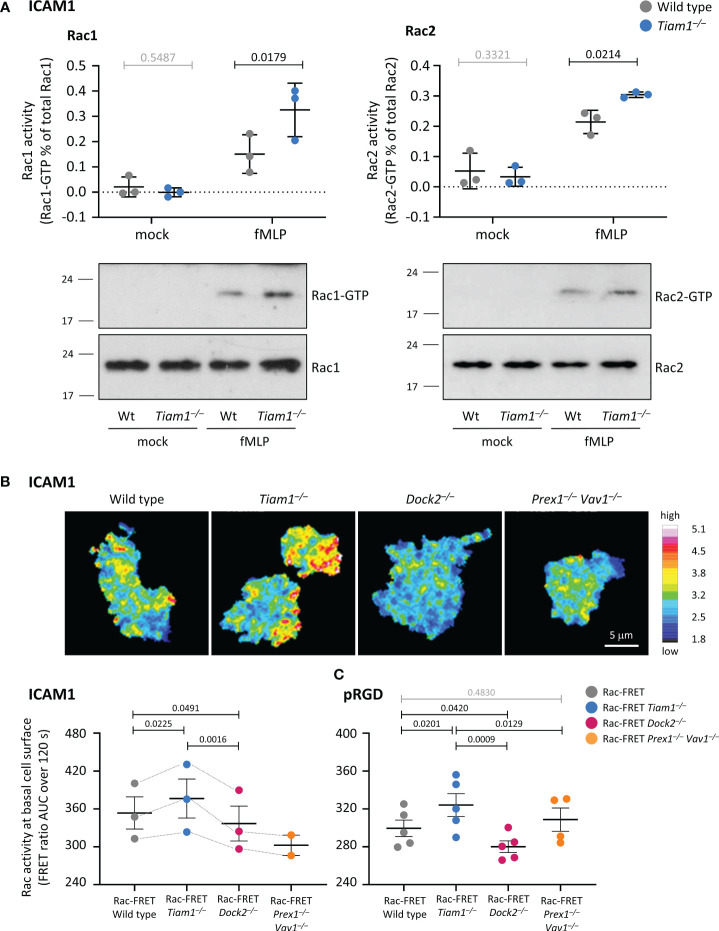
Tiam1 limits Rac activity in adherent neutrophils. **(A)** Rac activity (Pak-CRIB) in neutrophils adhering to ICAM1. Wild type (grey symbols) and *Tiam1^–/–^* (blue symbols) neutrophils were primed with 50 ng/ml GM-CSF, 20 ng/ml TNFα for 45 min at 37˚C, plated onto ICAM1 for 60 min, and stimulated with 1.5 μM fMLP, or mock-stimulated, for 1 min. Rac1 and Rac2 activities (GTP-loading) in adherent cells were determined by PAK-CRIB assay. Western blots are from one experiment representative of 3; 2% of the total lysate was loaded in the lower panels. Data are mean ± SEM of 3 independent experiments; each dot is the mean of one experiment. Statistics are two-way ANOVA with Sidak’s multiple comparisons test. **(B)** Rac activity in live neutrophils adhering to ICAM1. Neutrophils from Rac-FRET mice which express the Raichu-Rac reporter (Rac-FRET Wild type, grey symbols), or from Rac-FRET mice deficient in Tiam1 (blue), Dock2 (pink) or Prex1/Vav1 (orange) were primed as in **(A)**, plated onto ICAM1-coated ibidi slides in the presence of 0.75 µM fMLP for 60 min, and then live-imaged by ratiometric TIRF-FRET imaging for 2 min at a frame interval of 5 s. Representative stills from **Supplementary Movie 3**. The pseudo-colour scale depicts high Rac activity (high FRET ratio) at the basal cell surface in white/red and low Rac activity in blue. Quantification of Rac activity (FRET ratio) at the basal cell surface as mean AUC ± SEM of 3 independent experiments (2 for Rac-FRET *Prex1^–/–^ Vav1^–/–^*). **(C)** Rac activity in live neutrophils adhering to pRGD. Neutrophils from Rac-FET mice were treated and imaged as in **(B)** except on pRGD-coated ibidi slides. Rac activity is the mean AUC ± SEM of 5 independent experiments (4 for Rac-FRET *Prex1^–/–^ Vav1^–/–^*). Statistics in **(B, C)** are one-way ANOVA on log-transformed data with Tukey’s multiple comparisons test. **(A–C)** P-values in black denote significant differences, p-values in grey are non-significant.

First, we tested Rac activity in neutrophils in suspension by Pak-CRIB pull down. Both Rac1 and Rac2 were robustly and dose-dependently activated upon fMLP stimulation, as expected, but there was no difference between wild type and *Tiam1^–/–^* cells (**Supplementary Figure 9**), consistent with the normal ROS production and degranulation in suspension. In neutrophils adhering to ICAM1, fMLP stimulation also induced robust activation of Rac1 and Rac2. However, both these Rac activities were higher in *Tiam1^–/–^* neutrophils than in wild type (**Figure 8A**). Hence, Tiam1 paradoxically limits the chemoattractant-dependent activation of Rac in adherent neutrophils, rather than promoting it as would be expected from a Rac-GEF.

To investigate Rac activity in a different manner, we compared neutrophils from Rac-FRET mice that express a Raichu-Rac FRET reporter for Rac activity to Rac-FRET neutrophils deficient in Tiam1, Dock2 or Prex1/Vav1 (25). Neutrophils were plated onto ICAM1 or pRGD in the presence of fMLP and were live-imaged by ratiometric TIRF-FRET imaging to visualise Rac activity at the basal cell surface which touches the substrate (**Figures 8B, C**; **Supplementary Movie 3**). Rac activity (FRET ratio) was reduced in Rac-FRET *Dock2^–/–^* neutrophils under both conditions, and was either normal or reduced in Rac-FRET *Prex1^–/–^ Vav1^–/–^* cells (**Figures 8B, C**), as expected (25). In contrast, Rac activity was increased in Rac-FRET *Tiam1^–/–^* cells, both on ICAM1 and pRGD (**Figures 8B, C**), in line with the results obtained by Pak-CRIB pull down assay. The same increased Rac activity was seen when we plated Rac-FRET *Tiam1^–/–^* neutrophils on glass instead of ICAM1 or pRGD (**Supplementary Figure 10A, Movies 4–6**), and when we used widefield ratiometric FRET imaging of cells on ICAM1 instead of TIRF-FRET in order to image the whole cell (**Supplementary Figure 10B**). Furthermore, Rac activity was also increased when *Tiam1^–/–^* neutrophils were plated on M18/2 anti-CD18 antibody, which activates β2 integrins (49, 50) (**Supplementary Figure 10C**). In contrast, Rac activity was decreased in Rac-FRET *Dock2^–/–^* cells throughout, as expected (**Supplementary Figure 10A–C, Movies 4–6**). Hence, Tiam1 limits Rac activity in adherent neutrophils, rather than being required for the activation of Rac as would be expected for a Rac-GEF.

Furthermore, we previously established that peak Rac activity oscillates between the leading edge and uropod of migrating neutrophils plated on glass (44). We investigated if Tiam1 may contribute to these waves in a pilot experiment, by determining the localisation of peak Rac activity along the central longitudinal axis of Rac-FRET *Tiam1^–/–^* neutrophils during their migration towards a micropipette containing fMLP. In wild type neutrophils, Rac activity oscillated between leading edge and uropod every 7 s, whereas these waves were shorter (6 s) in *Tiam1^–/–^* cells, although the distance migrated and likelihood to migrate were unaffected under these conditions (**Supplementary Figure 11**). Moreover, peak Rac activity was lost from the leading edge of *Tiam1^–/–^* neutrophils, with both the amount of time and number of events of peak activity localising within 0.8 μm of the leading edge being reduced, whereas Rac activity localisation at the uropod was normal (**Supplementary Figure 11**). These result warrant detailed analysis elsewhere in the future, but they do suggest that Tiam1 may regulate Rac activity waves and generate Rac activity at the leading edge during neutrophil migration.

### Tiam1 controls which proteins interact with Rac during neutrophil adhesion to ICAM1

**Figure 9:**
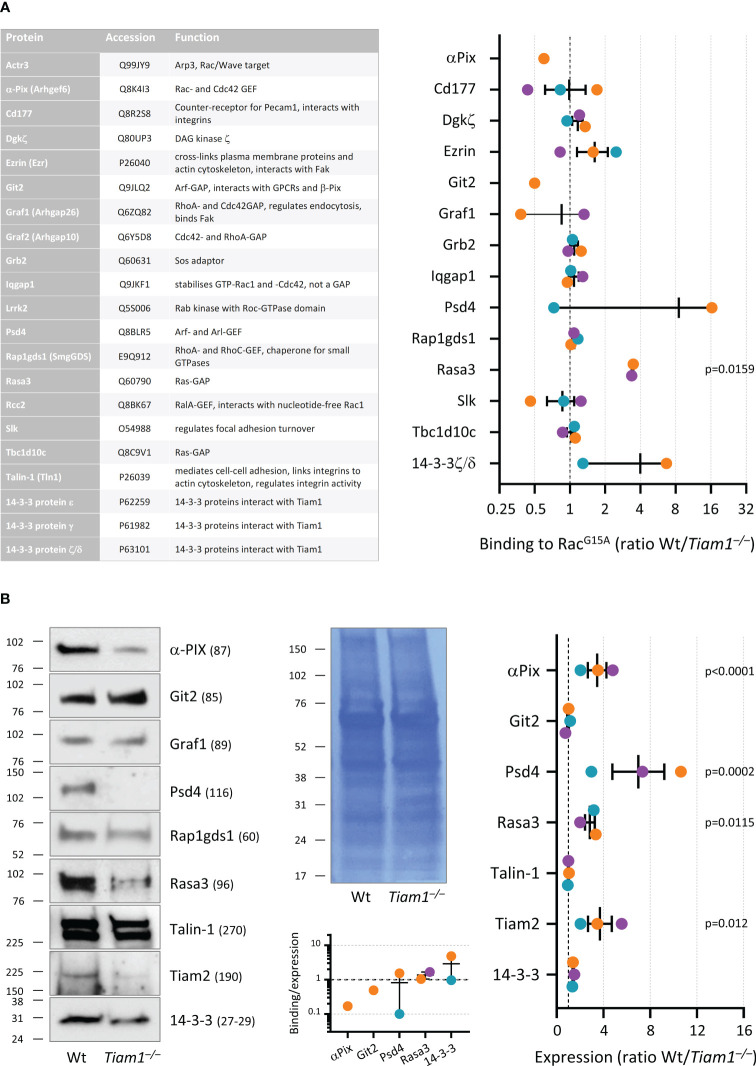
Tiam1 controls the expression of small GTPase and cytoskeletal regulators, and the association of Rasa3 with Rac in neutrophils adhering to ICAM1. Wild type and *Tiam1^–/–^* neutrophils were allowed to adhere to 50 mm glass coverslips coated with ICAM1 during priming with 50 ng/ml GM-CSF, 20 ng/ml TNFα for 50 min at 37°C, 5% CO_2_. DFP was added for 10 min before neutrophils were stimulated with 1.5 µM fMLP for 1 min and then lysed. The cleared supernatants (total lysates) were incubated with immobilised Rac1^G15A^ to isolate proteins that interact with nucleotide-free Rac. **(A)** Targeted mass spectrometry was performed for 21 proteins found to bind Rac1^G15A^ (left). 14 proteins could be compared between wild type (Wt) and *Tiam1^–/–^* samples, 12 proteins in at least 2 out of 3 independent experiments (right). Wt/*Tiam1^–/–^* ratios are mean ± SEM or range, as appropriate, of 2-3 independent experiments; coloured dots depict the different experiments. Statistics are one-way ANOVA with Dunnett’s multiple comparisons test on log-transformed ratios. **(B)** Total lysates from the experiments in **(A)** were western blotted with the indicated antibodies. Representative western blots and coomassie loading control are shown. Blots of selected proteins were quantified by densitometry (right). Wt/*Tiam1^–/–^* ratios are expressed as mean ± SEM of 3 independent experiments; statistics are two-way ANOVA with Sidak’s multiple comparisons test on raw band intensities. Bottom panel: The Wt/*Tiam1^–/–^* Rac1^G15A^ binding ratio is expressed as a function of the Wt/*Tiam1^–/–^* expression level for the indicated proteins.

Searching for mechanisms that might underlie the paradoxical increases in Rac and integrin activity, adhesion, polarity, and migration in adherent *Tiam1^–/–^* neutrophils, we hypothesised that other Rac-GEFs likely took over the role of Tiam1 and overcompensated. To test this hypothesis, we used the Rac mutant Rac1^G15A^, which stabilises the transient nucleotide-free state that occurs during the activation of Rac and has high affinity for active Rac-GEFs (52). We stimulated wild type and *Tiam1^–/–^* neutrophils adhering to ICAM1 with fMLP, affinity-purified proteins that interact with Rac1^G15A^ from lysates, and identified them by mass spectrometry. Preliminary analysis identified 501 proteins in at least one sample (**Supplementary Table 1**). We selected 21 proteins based on the literature, for their ability to regulate small GTPases and/or cytoskeletal dynamics, or to interact with Tiam1, and analysed them further by targeted proteomics. We could compare 14 of these proteins between the genotypes, and 12 in at least two of three experiments (**Figure 9A**). The Rac-and Cdc42-GEF α-Pix (63) appeared to bind Rac1^G15A^ more strongly in *Tiam1^–/–^* neutrophils than wild type (Wt/Ko ratio = 0.6), as did the Arf-GAP Git2, an interactor of GPCRs and β-Pix (64) (Wt/Ko ratio = 0.5). The stronger association of α-Pix with Rac1^G15A^ might explain the higher Rac activity in *Tiam1^–/–^* neutrophils. However, α-Pix and Git2 were only detected in one experiment, so we cannot draw firm conclusions. In contrast, the dual Ras- and Rap-GAP Rasa3 (65, 66) consistently interacted with Rac1^G15A^ more strongly in wild type than *Tiam1^–/–^* neutrophils (3.4-fold). The Arf- and Arl-GEF Psd4 (67, 68) and the adaptor protein 14-3-3ζ/δ, which bridges Tiam1 to β1 integrins (69), followed a similar trend but this did not reach significance due to considerable variability (**Figure 9A**). The other 9 proteins bound Rac1^G15A^ equally between wild type and *Tiam1^–/–^*. They comprised Cd177, a counter-receptor for Pecam1 that interacts with integrins (70); the diacylglycerol kinase Dgkζ (71); Ezrin, which crosslinks transmembrane and cytoskeletal proteins and interacts with focal adhesion kinase (72); the RhoA- and Cdc42-GAP Graf1 (73); the Sos adaptor Grb2 (74); the adaptor protein Iqgap1 which stabilises GTP-bound Rac1 and Cdc42 (75); the chaperone Rap1gds1 (also known as SmgGDS) which acts as an atypical RhoA and RhoC-GEF (76); the protein kinase Slk, which regulates focal adhesion turnover (77); and the Ras-GAP Tbc1d10c (78). Hence, Tiam1 controls the association of the Ras-GAP Rasa3, and possibly also of α-Pix, Git2, Psd4 and 14-3-3ζ/δ with nucleotide-free Rac.

Finally, we evaluated the total cellular levels of some of the proteins we identified (**Figure 9B**). α-Pix was 3.5-fold higher in wild type neutrophils than *Tiam1^–/–^*, suggesting that Tiam1 promotes α-Pix expression or stability, despite possibly limiting α-Pix binding to Rac1^G15A^. In contrast, Git2 was expressed normally. Rasa3 was 2.9-fold higher in wild type neutrophils and Psd4 7.0-fold higher, which likely explains their association with Rac1^G15A^. 14-3-3 expression was normal, despite potentially stronger binding to Rac1^G15A^ in wild type, suggesting Tiam1 may mediate 14-3-3ζ/δ binding to Rac. Tiam2, which was not detected among Rac1^G15A^ interactors, was expressed 3.7-fold higher in wild type cells, suggesting that this GEF did not compensate in *Tiam1^–/–^* cells. Talin, a master regulator of adhesion that links integrins and focal adhesions to the cytoskeleton (79), and co-purified with Rac1^G15A^ in some samples, was expressed normally. Finally, Graf1 and Rapgds1 also appeared to be expressed normally, although we did not formally quantify them.

**Figure 10:**
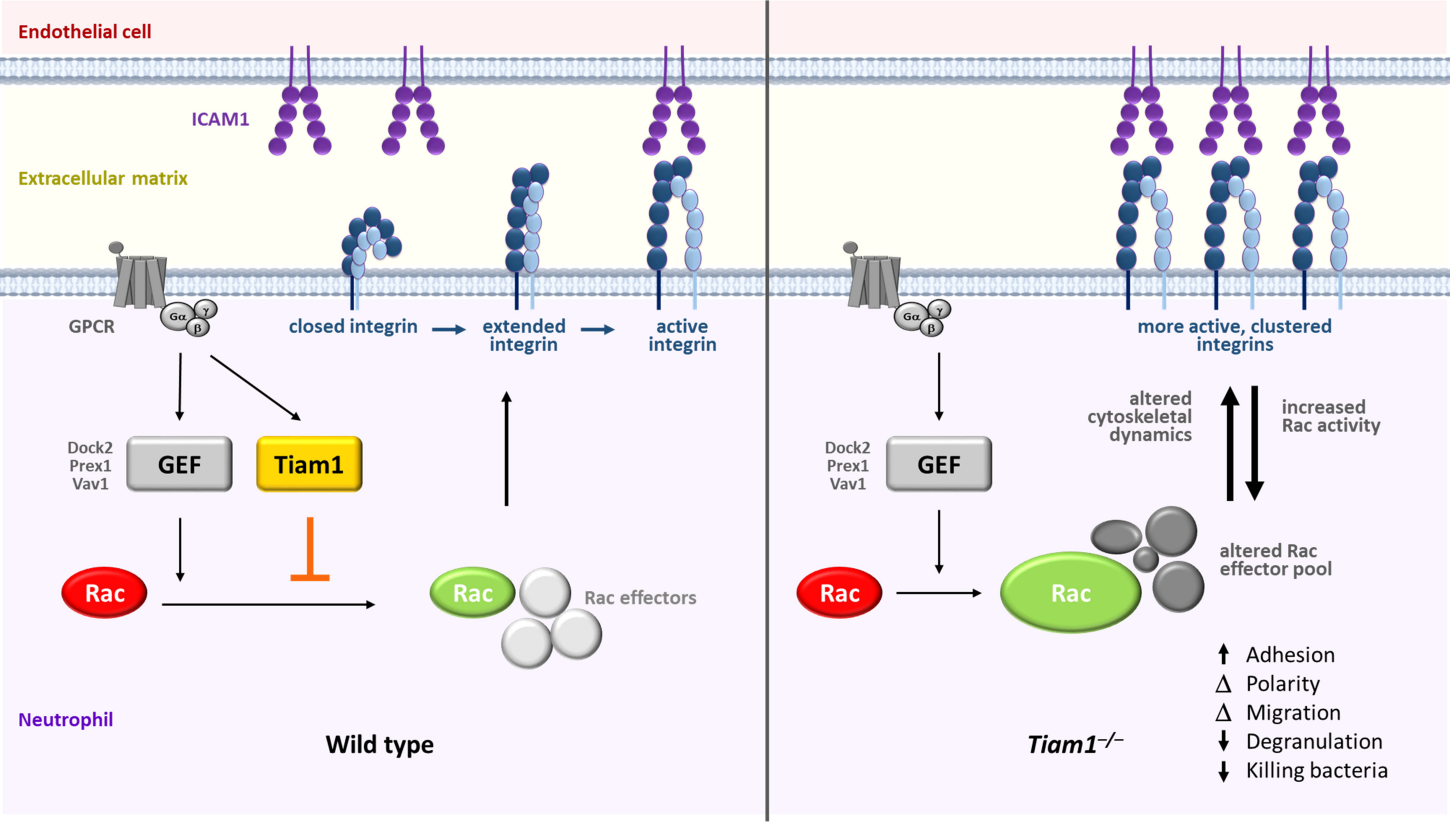
Model: Tiam1 controls neutrophil responses in a different manner to other Rac-GEFs. Several neutrophil Rac-GEFs, including Prex1, Vav1 and Dock2 are required for the activation of Rac in response to the stimulation of GPCRs, such as fMLP and CXCL1 receptors. Prex1 and Vav1 were previously shown to control integrin inside-out signalling, leading to the activation of β2 integrins which bind to ICAM1, thereby enabling neutrophil adhesion and migration. We showed here that the Rac-GEF Tiam1 acts differently, by limiting Rac activity in neutrophils adhering to ICAM1, rather than stimulating Rac activity as would be expected for a Rac-GEF (left panel). Tiam1 deficiency (right panel) increases Rac activity, alters the pool of regulators of small GTPases and cytoskeletal dynamics and their association with Rac, and increases the activity and avidity of β2 integrins. The latter shows that Tiam1 limits integrin inside-out signalling. This may underlie the de-regulated focal complex structures and cytoskeletal dynamics, neutrophil adhesion, polarisation and migration seen in Tiam1-deficient cells. The increased Rac activity in adherent Tiam1-deficient cells suggests that Tiam1 regulates integrin outside-in signalling as well as inside-out signalling. In addition to β2 integrins, Tiam1 also seems to control β1 integrins, with different effects on adhesion, polarisation and migration. Degranulation, NETs release and the capacity to kill bacteria are reduced in Tiam1-deficient neutrophils, as are neutrophil recruitment and the ability to clear bacteria in Tiam1-deficient mice *in vivo*. In contrast to its adhesion-dependent roles, Tiam1 is dispensable for Rac activation and degranulation in non-adherent neutrophils, and ROS production is normal.

In conclusion, Tiam1 promotes the expression of several regulators of small GTPase signalling and cytoskeletal dynamics, and it mediates the binding of Rasa3, and possibly also Psd4 and 14-3-3ζ/δ to Rac, as well as potentially limiting the association of α-Pix and Git2 with Rac. Although these data do not conclusively explain how Tiam1 limits Rac activity in adhering neutrophils, it seems likely that this extensive deregulation of relevant proteins does contribute. Together, our data show that Tiam1 is an important regulator of neutrophil responses, and particularly but not exclusively of adhesion-dependent responses, but it fulfils this role in a manner that differs fundamentally from those of other neutrophil Rac-GEFs, and indeed differently to what would be expected from any Rac-GEF (**Figure 10**).

## Discussion

Neutrophil Rac-GEFs such as Prex1, Vav1, and Dock2 activate the small GTPase Rac to mediate neutrophil responses. Here, we show that Tiam1 also controls neutrophils, but in a fundamentally different manner. Paradoxically, Tiam1 limits the chemoattractant-stimulated activation of Rac and of β2 integrins in adherent neutrophils, instead of promoting them as other Rac-GEFs do. Tiam1 controls the expression of GTPase and cytoskeletal regulators, and alters the pool of proteins that associate with Rac. It regulates focal complexes, cytoskeletal dynamics, adhesion, migration, degranulation, and NETs release in adherent neutrophils, as well as controlling cell morphology and bactericidal capacity both in adherent neutrophils and in suspension. *In vivo*, Tiam1 controls neutrophil recruitment during aseptic peritonitis and the clearance of pulmonary *S. pneumoniae* infection.

The most astonishing *Tiam1^–/–^* phenotype was the increased fMLP-stimulated Rac activity in adherent neutrophils. We are not aware of other instances of GEF deficiencies causing increased GTP-loading of GTPases, so the mechanism could not be inferred. An obvious explanation would be that another Rac-GEF overcompensated, or that negative regulators such as Rac-GAPs were lost. However, no Rac-GEF bound more to Rac1^G15A^ in *Tiam1^–/–^* cells, and no Rac-GAPs less. A possible exception was α-Pix, which bound Rac1^G15A^ more in *Tiam1^–/–^* cells when detectable, despite consistently higher expression in wild type. Like α-Pix, the Arf-GAP Git2 also bound Rac1^G15A^ more strongly in *Tiam1^–/–^* neutrophils when detectable, despite normal expression levels. Many instances of crosstalk between Arf and Rac GTPases have been described (64, 80, 81), including in neutrophils (82), so possibly Tiam1-deficiency impacted Arf activity indirectly, in turn leading to Rac activation. The dual Ras- and Rap-GAP Rasa3 bound Rac1^G15A^ more in wild type neutrophils, in line with its stronger expression. Rasa3 binding to Rac has not been reported before. Conceivably, increased Ras and Rap activities would feed-forward to Rac through Rac-GEFs sensitive to these pathways, such as Prex1, Tiam2, and Vav. The Arf- and Arl-GEF Psd4 also appeared to bind Rac more in wild type cells, in line with its higher expression, as did the adaptor protein 14-3-3ζ/δ, despite normal 14-3-3 expression. The latter may contribute to the altered focal complex structures in adherent *Tiam1^–/–^* neutrophils. Furthermore, we detected Talin as a Rac1^G15A^ interactor, although not in sufficient samples for comparison between genotypes. Tiam1 interacts directly with Talin, which binds and activates integrins and stabilises cell/cell adhesions (79). Together, Talin, Tiam1, and Par polarity complex regulate Rac1 activity in adhesion turnover (83). Altogether, the deregulated association of regulators of small GTPases, integrin activity, and focal complexes with Rac may provide an explanation for the altered cytoskeletal dynamics and effector responses in *Tiam1^–/–^* neutrophils.

Tiam1 deficiency increased the expression levels of α-Pix, Psd4, Rasa3, and Tiam2. It is possible that Tiam1 regulates the expression of these proteins directly. Unlike most Rac-GEFs, Tiam1 has a nuclear localisation signal and can interact with transcription factors such as c-Myc and RORγt, and with the transcriptional co-activators Yap/Taz in the nucleus, to either repress or stimulate transcription (41, 84, 85). The mechanisms through which Tiam1 controls protein levels in neutrophils require further investigation.

The main integrin types expressed in neutrophils are β1 and β2 (55), although β3, β5, and β7 are also detectable (42). Our study shows that Tiam1 limits the CXCL1-stimulated increase in β2 integrin affinity and avidity. It would be interesting to investigate in further detail if Tiam1 differentiates between the two β2-integrins LFA-1 and Mac-1. CXCL1 is the major chemokine responsible for the activation of β2 integrins during neutrophil extravasation *in vivo* in mice (86), so this is likely relevant in the impaired neutrophil recruitment observed in *Tiam1^–/–^* mice during peritonitis. Comparison of ICAM1 to pRGD and fibrinogen suggested that Tiam1 also controls β1 integrins, as polarisation and random migration of *Tiam1^–/–^* neutrophils on pRGD were increased, but not β3 integrins, as migration on fibrinogen was normal. Furthermore, β1 and β2 integrins activate distinct signalling pathways, which may explain how Tiam1 affects cell behaviours on ICAM1 and pRGD so differently. However, the effects of Tiam1 on β1 integrin activity remain to be confirmed, as it is not possible to infer integrin specificity from adhesion and migration on a ligand alone. In general, it is not trivial to ascertain integrin specificity in primary mouse neutrophils, due to limited availability of reagents, so this may have to be done by downregulating Tiam1 in human neutrophil-like cell lines.

*Tiam1^–/–^* neutrophils adhered to ICAM1 more under some conditions, but chemotaxis was reduced. This is unsurprising, because neutrophils require finely balanced levels of adhesion, spreading, and polarisation to generate the forces required for migration (1, 87). The increased adhesion, reduced polarity, and reduced focal complex size can explain the decreased migration speed of *Tiam1^–/–^* neutrophils during chemotaxis on ICAM1, whereas the increased polarity and number of focal complexes may underlie the increased random migration on pRGD. It remains unclear whether adhesion strength is affected by Tiam1 deficiency. Our tests of adhesion and migration under shear stress indicated that adhesion strength is unaffected, as primed *Tiam1^–/–^* neutrophils adhered normally to ICAM1 both before and after the application of shear flow. However, we cannot exclude that adhesion strength is affected under other conditions. Rigorous measurement of adhesion strength would require biophysical approaches such as atomic force microscopy. The finding that fMLP-stimulated chemotaxis was reduced whereas CXCL1-stimulated chemotaxis was increased in *Tiam1^–/–^* neutrophils in the transwell assay showed an interesting pathway dependence that requires detailed future investigation. The increased CXCL1-dependent integrin affinity and avidity, and the elevated chemotaxis of *Tiam1^–/–^* neutrophils *in vitro*, might underlie the altered recruitment *in vivo*. Furthermore, as Tiam1 affected the morphology of basal neutrophils in suspension as well as that of adherent neutrophils, it likely plays roles in cytoskeletal structure beyond controlling GTPase and integrin signalling, possibly linked to its functions in protein expression discussed here-above.

Neutrophil recruitment during aseptic peritonitis was impaired in *Tiam1^–/–^* mice, whereas recruitment appeared normal overall during *S. pneumoniae* infection. The time points chosen differed between the two models, so that each was assessed under conditions where neutrophil recruitment is maximal (13, 15). Histological analysis revealed that recruitment was altered in the *S. pneumoniae*-infected lungs of *Tiam1^–/–^* mice. During the acute phase of infection tested, neutrophils are most concentrated within the interstitial space between vasculature and airway epithelium. This interstitial localisation was reduced in *Tiam1^–/–^* mice, suggesting that Tiam1 either controls the route taken by neutrophils during extravasation, as it does for T lymphocyte transmigration (39), or affects the kinetics of recruitment, with reduced dwell-time in the interstitium. We did not observe a corresponding increase of neutrophils elsewhere, to account for the overall normal numbers. We presume that more *Tiam1^–/–^* neutrophils might accumulate in the comparatively vast alveolar epithelium, as neutrophil extravasation in the inflamed lung primarily occurs at capillaries in the interalveolar septum (88). Our failure to detect this likely reflects a limitation of assessing H&E-stained lung histology sections, which favours detection of neutrophils where they are most concentrated, in regions encompassing interstitium and the adjacent vasculature and airway epithelium, but is not suited for quantification of neutrophils throughout the relatively vast alveolar tissue. MPO IHC allows for more sensitive detection of neutrophils throughout the lung, but only prior to the degranulation of azurophil granules. The latter approach did not show significant changes, only a trend for increased MPO signal in *S. pneumoniae*-infected *Tiam^–/–^* mice, and as MPO degranulation was impaired in *Tiam1^–/–^* neutrophils *in vitro*, it was difficult to draw conclusions regarding recruitment from the MPO IHC. Ly6G staining may be an alternative. In general, it is hard to document neutrophil transmigration in fixed tissue, as transmigration is a much faster process than adhesion and crawling on the endothelium, so is a relatively rare event. Unequivocal assessment of *Tiam^–/–^* neutrophil extravasation would require intravital microscopy.

Rac activation was increased and degranulation reduced in adherent *Tiam1^–/–^* neutrophils but normal in suspension, whereas cell morphology and bacterial killing were affected both in adherent cells and in suspension. Changes in morphology, degranulation, and NETs release all require cytoskeletal rearrangements, and it seems likely that the altered F-actin dynamics in *Tiam1^–/–^* neutrophils, particularly the enhanced cortical F-actin ring, underlie these impairments. ROS production was normal both in adherent cells and in suspension, so clearly the increased Rac2-activity was insufficient to enhance this response. We measured ROS production in response to various soluble and particulate stimuli in the presence of HRP and cell-permeable luminol, to capture both extra- and intracellular ROS, and to include all manner of ROS (O_2_^-^, H_2_O_2_, HOCl) (89). It might be interesting to investigate extracellular and intraphagosomal ROS separately. It was intriguing that NET release was reduced despite normal ROS production, as NETs are largely ROS-dependent. However, NET release requires cytoskeletal remodelling and degranulation in addition to ROS, and these responses were impaired. The reduced degranulation and NET release can explain the impaired capacity of *Tiam1^–/–^* neutrophils to kill bacteria. It would be interesting to also investigate the role of Tiam1 in phagocytosis in the future. The conditions of bacterial titre and early timepoint of the *S. pneumoniae* infection we used *in vivo* results in mild acute inflammation, although the infection would prove fatal if allowed to progress for several more days (13, 90). We did not detect significant degranulation or NETs under these relatively mild conditions, but it seems likely that impairments in low levels of these responses contribute to the reduced bacterial clearance in *Tiam1^–/–^* mice, together with the altered distribution of neutrophils in the lung. The fact that Tiam1 affects some responses in adherent cells but not in suspension may underlie the impaired bacterial clearance in lavaged and perfused lung tissue of *Tiam1^–/–^* mice but normal clearance in the airways. In the former, leukocytes and bacteria adhere firmly enough to withstand perfusion, whereas in the latter they can be lavaged-out.

The phenotype of *Tiam1^–/–^* neutrophils is similar but not identical to that resulting from knockdown of Tiam2 in haematopoietic stem cells differentiated *in vitro* into neutrophils (43). The vinculin-containing focal complexes in wild type neutrophils on pRGD were quite indistinct, as expected for neutrophils (61). However, in *Tiam1^–/–^* neutrophils, these structures resembled more the focal adhesions observed in other cell types. Similar structures were found in the neutrophil-like cells depleted of Tiam2 (43). The Tiam2-depleted cells also showed impaired fMLP-stimulated migration and increased F-actin, but on fibrinogen, where we did not observe defects in *Tiam1^–/–^* cells. Furthermore, Tiam2-depleted cells showed exaggerated F-actin at the leading edge but not the de-localised F-actin at the uropod we see in *Tiam1^–/–^* cells. Therefore, it seems that Tiam1 and Tiam2 play similar but non-redundant roles in neutrophils, potentially by controlling different sets of integrins. Tiam1 and Tiam2 have a similar domain structure, but some of their domains (e.g. PDZ) show low sequence homology, they have distinct subcellular localisations and interact with different sets of upstream regulators, and control different Rac-dependent responses, with Tiam2 in particular controlling the morphology of the nucleus in various cell types (33, 54, 91). It would be interesting to generate double-deficient neutrophils to investigate possible redundancy between the two GEFs.

Our analysis of spatiotemporal patterns of Rac activity suggests that Tiam1 may control the distribution of Rac activity during neutrophil chemotaxis. We showed recently that Dock2 generates Rac activity at the leading edge and uropod of chemotaxing neutrophils, but both Dock2 and Prex1/Vav1 were dispensable for the oscillations of Rac activity between the leading edge and uropod during migration (25). Data here suggest that peak Rac activity is lost from the leading edge *Tiam1^–/–^* cells and the oscillations of Rac activity between the leading edge and uropod were quicker. These data require future corroboration, but suggest that Tiam1 may not only regulate the amount of Rac activity differently to other GEFs, but also its distribution in space and time. We made attempts to determine the localisation of Tiam1 within the neutrophil by imaging. However, despite antibodies proving useful in other applications (92), they were not suitable in neutrophils (data not shown). Furthermore, it would be interesting to determine RhoA activity, which we would expect to decrease in *Tiam1^–/–^* neutrophils where Rac activity is increased. This is seen in many cell types and signalling contexts, and is mediated through several feedback pathways between Rac and Rho (93–96). Finally, Tiam1 is a large multidomain protein with many known scaffolding functions (33). It would be interesting to test how much the Rac-GEF activity of Tiam1 contributes to its regulation of neutrophil responses, for example through the use of mice with catalytically inactive Tiam1.

In conclusion, our study has shown that Tiam1 regulates neutrophil focal complexes, actin cytoskeletal dynamics, adhesion, polarisation, migration, degranulation, NETs release, and killing of bacteria, as well as controlling neutrophil recruitment and clearance of bacteria *in vivo*. However, contrary to expectations, Tiam1 limits the chemoattractant-stimulated activation of Rac1, Rac2, and β2 integrins instead of promoting them. Tiam1 determines the expression level of regulators of small GTPases and cytoskeletal dynamics and the association of a subset of these with Rac, and we propose that this underlies the curious effects of Tiam1 on Rac activity and on Rac-dependent neutrophil responses.

## Data availability statement

The data presented in the study are deposited in the PRIDE repository, accession number PXD046791.

## Ethics statement

The animal study was approved by Babraham Animal Welfare Ethical Review Body. The study was conducted in accordance with the local legislation and institutional requirements.

## Author contributions

KH, MB and PM contributed equally to this work. KH, MB, PM, SC, A-KJ, CP, PI, MS and LC planned, conducted and analysed experiments and made graphs. KH, MB and PM wrote part of the manuscript. DO planned and conducted the mass spectrometry and its data analysis. HO developed image analysis macros and helped with image analysis. SW planned and helped with FRET imaging and image analysis. RW planned and helped with Imagestream experiments. AS-P planned experimental design and helped with statistical analysis. YF provided the Dock2-deficient mouse strain and advice on Dock2 signalling. AM provided the Tiam1-deficient mouse strain and advice on Tiam1 signalling. HW planned and supervised the project, obtained the funding and wrote the manuscript. All authors reviewed the manuscript.
