# Supplementary material for: The Rac-GEF Tiam1 controls integrin-dependent neutrophil responses
Source: Front Immunol. 2023 Nov 21;14:1223653. doi: 10.3389/fimmu.2023.1223653 (PMC10703174; doi:10.3389/fimmu.2023.1223653)
Supplement: Supplementary file 2 [file DataSheet_1.pdf]

Supplementary Material

The Rac-GEF Tiam1 controls integrin-dependent neutrophil responses

Kirsti Hornigold[1,2], Martin J Baker[1,2,3], Polly A Machin[1,2], Stephen A Chetwynd[2], Anna-Karin Johnsson[2,4], Chiara Pantarelli[2], Priota Islam[2], Melanie Stammers[5], Laraine Crossland[2], David Oxley[6], Hanneke Okkenhaug[7], Simon Walker[7], Rachael Walker[8], Anne Segonds-Pichon[9], Yoshinori Fukui[10], Angeliki Malliri[3], Heidi C E Welch[2,*]

[1] These authors contributed equally to this work and share first authorship.

[2] Signalling Programme, Babraham Institute, Babraham Research Campus, Cambridge, UK
[3] Cell Signalling Group, Cancer Research UK Manchester Institute, University of Manchester, Alderley Park, Macclesfield, UK
[4] Present address: Division of Immunology and Allergy, Department of Medicine Solna, Karolinska Institute, and Karolinska University Hospital, Clinical Immunology and Transfusion Medicine, Stockholm, Sweden
[5] Roving Researcher, Babraham Institute, Babraham Research Campus, Cambridge, UK
[6] Mass Spectrometry Facility, Babraham Institute, Babraham Research Campus, Cambridge, UK
[7] Imaging Facility, Babraham Institute, Babraham Research Campus, Cambridge, UK
[8] Flow Cytometry Facility, Babraham Institute, Babraham Research Campus, Cambridge, UK
[9] Bioinformatics Facility, Babraham Institute, Babraham Research Campus, Cambridge, UK
[10] Division of Immunogenetics, Department of Immunobiology and Neuroscience, Medical Institute of Bioregulation, Kyushu University, Fukuoka, Japan

*** Correspondence:**
Heidi Welch
heidi.welch@babraham.ac.uk

Content:

Supplementary Figures 1-11 and Legends

Legends to Supplementary Movies 1-6

Supplementary Table 1: separate Excel file

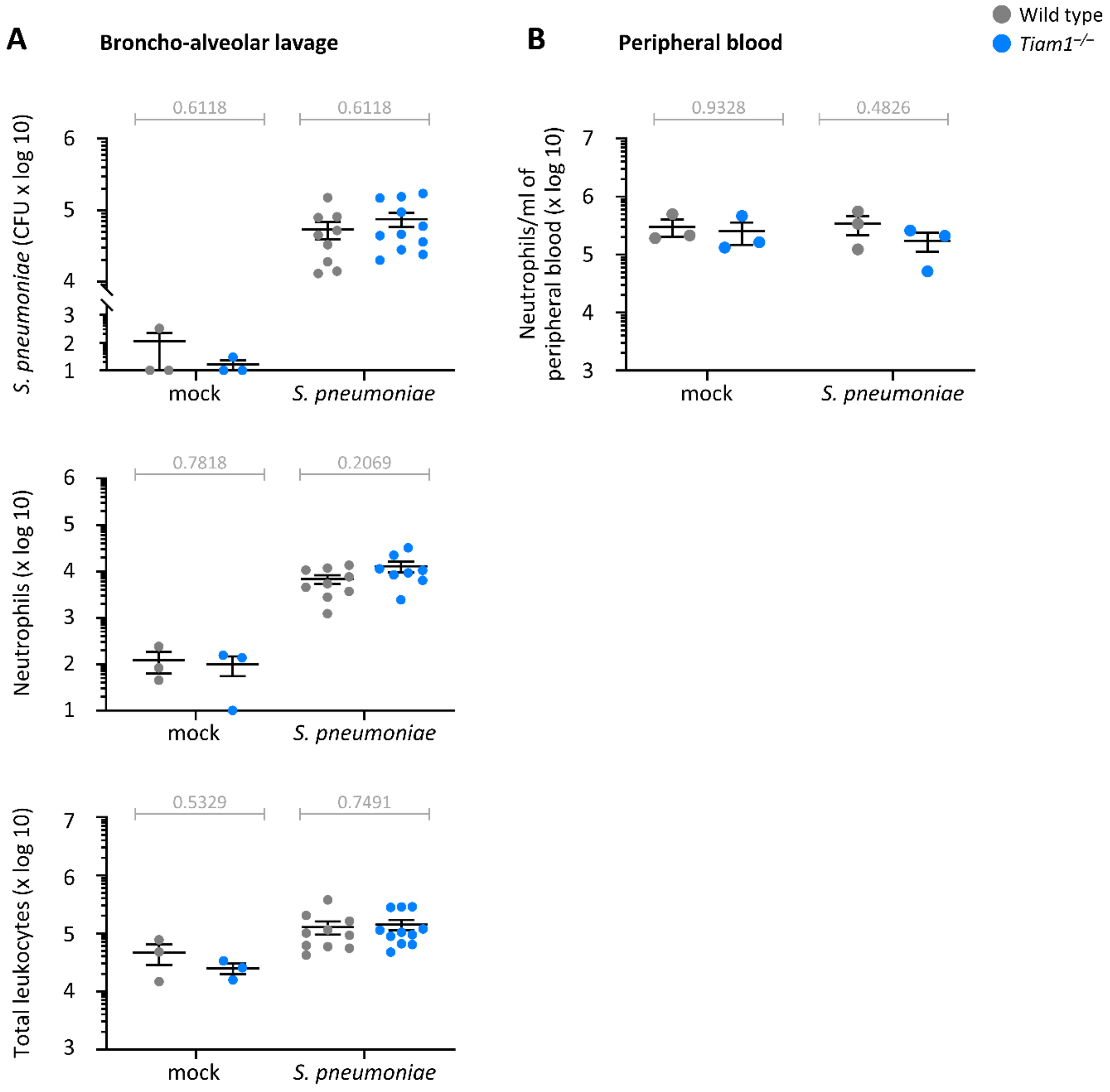

Supplementary Figure 1. Tiam1 is dispensable for clearing *S. pneumoniae* from the airways. Wild type (grey symbols) and (blue symbols) mice were infected *i.n.* with 2 x 10^6 CFU *S. pneumoniae*, or were mock-treated, and culled 6 h later. **(A)** Bronchoalveolar lavage (BAL) was performed before lungs were perfused, excised and homogenized. The BAL fluid was cultured for enumeration of *S. pneumoniae* CFU (top panel), or stained to identify neutrophils ($Ly6G^{hi}$, $CD11b^{hi}$; middle panel) and total leukocytes ($CD45^{+}$; bottom panel) by flow cytometry. Data are mean ± SEM of mice pooled from 3 independent experiments, with 1 mock and 3-4 *S. pneumoniae* treated mice per experiment; each dot represents one mouse. The data are from the same experiments as those shown in Figure 1D. Statistics are one-way AVOVA on log-transformed data followed by pairwise comparisons with Holm-Sidak's multiple comparisons test. **(B)** Neutrophils in the peripheral blood were analysed by flow cytometry. Data are mean ± SEM of 3 mice; each dot represents one mouse. The data are from the same experiments as those shown in Figure 1E. Statistics are two-way AVOVA with Sidak's multiple comparisons test. P-values in grey are non-significant.

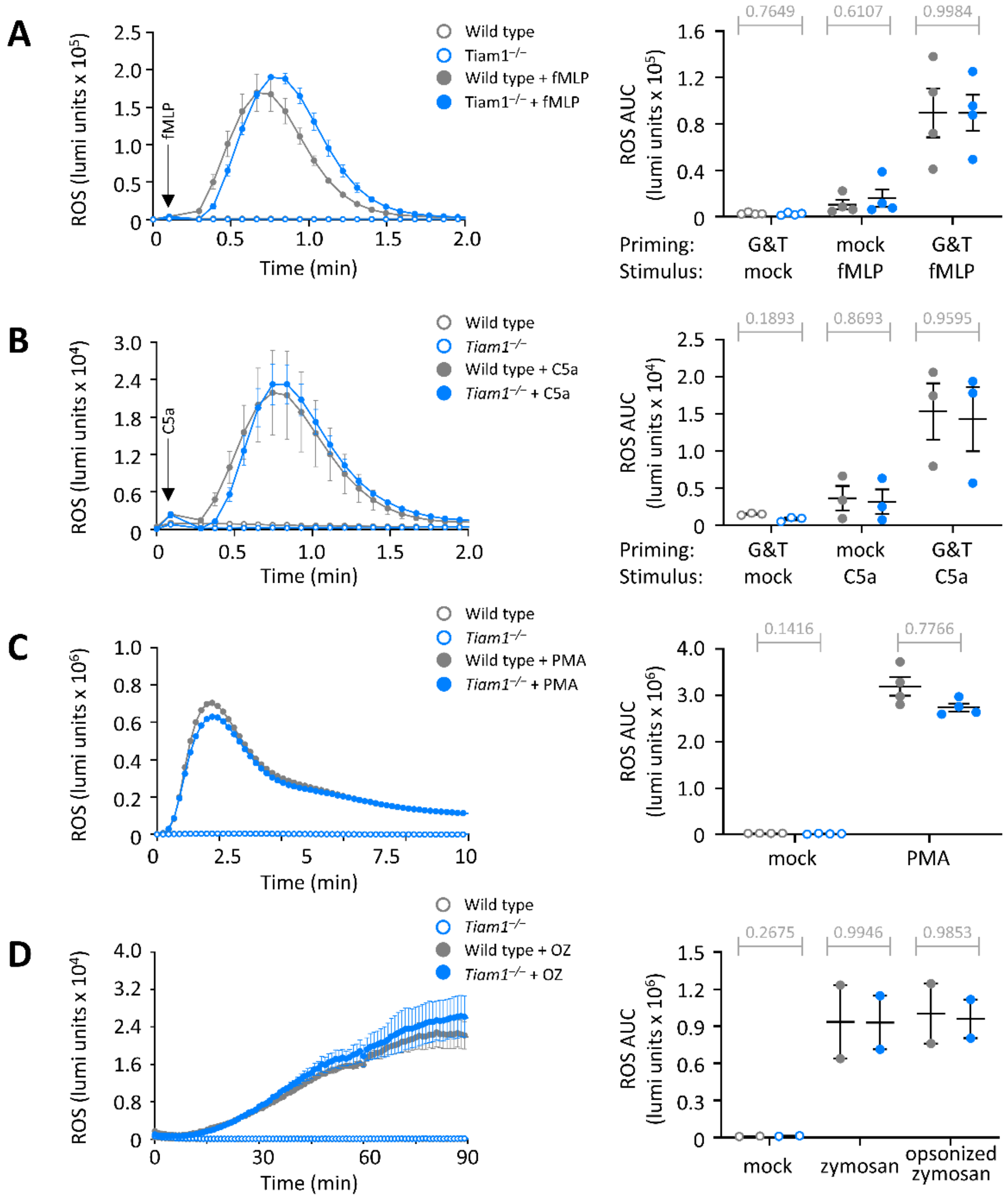

Supplementary Figure 2. Tiam1 does not regulate ROS production in neutrophils in suspension. ROS production was measured in real time in wild type (grey symbols) and *Tiam1*$^{-/-}$ (blue symbols) neutrophils in suspension by luminometer in the presence of luminol and HRP to assess intra-and extracellular ROS combined, in response to stimulation with (A) 3 μM fMLP, (B) 25 nM C5a, (C) 500 nM PMA, or (D) with unopsonised or serum-opsonised zymosan yeast particles (3 particles per neutrophil). Prior to the assay, neutrophils were either kept basal (on ice), primed with 100 ng/ml GM-CSF, 5 ng/ml TNFα for 1 h at 37˚C, or mock-primed, as indicated. Left: representative curves; arrows denote the time of addition of stimulus by injection port. Right: Data are mean AUC ± SEM of 4 independent experiments in (A) and (C), 3 in (B) and 2 in (D). Each dot is the mean of one experiment. Statistics are two-way ANOVA with Sidak's multiple comparison corrections; p-values in grey denote non-significant differences between genotypes.

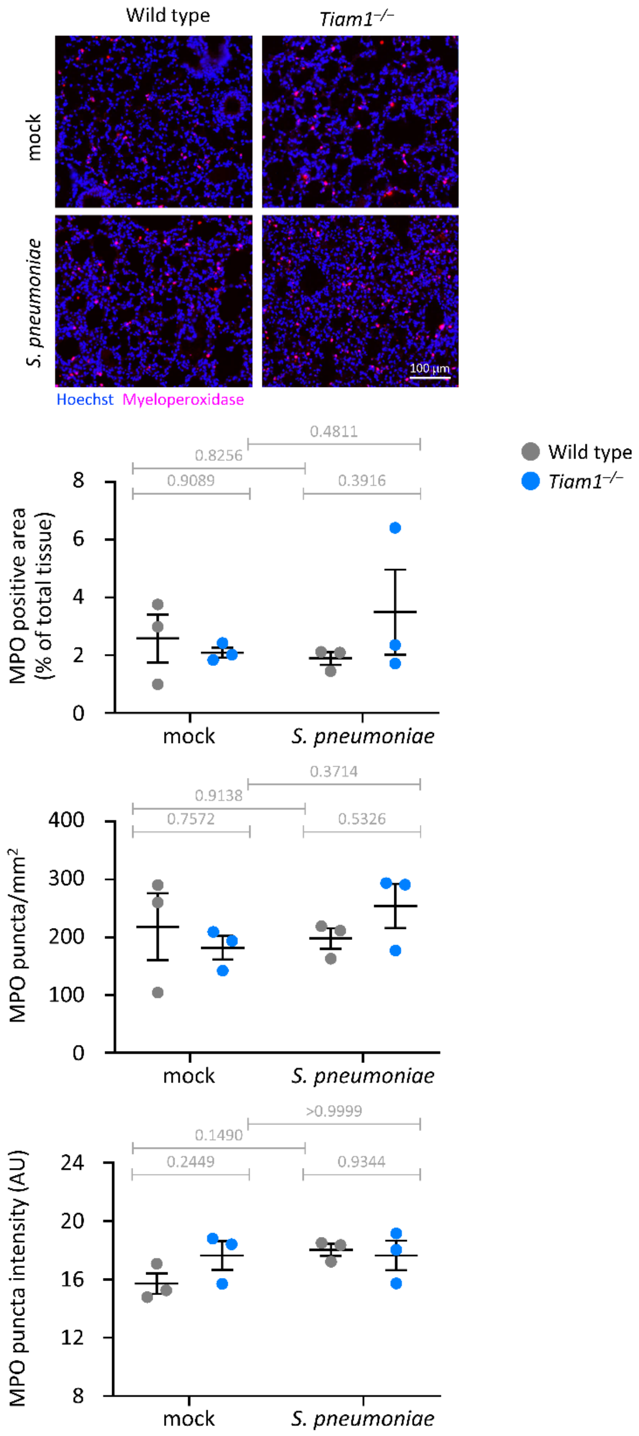

Supplementary Figure 3. Tiam1 does not regulate MPO levels in the *S. pneumoniae*-infected lung. MPO levels were measured in lung histology sections of the *S. pneumoniae*-infected and mock-infected wild type (grey symbols) and *Tiam1*$^{-/-}$ (blue symbols) mice shown in Figure 1E. Sections were analysed for the MPO positive tissue area, for the number of MPO positive puncta and for the fluorescence intensity of these puncta. Data are mean AUC ± SEM of 3 mice per condition; each dot represents one mouse. Statistics are two-way ANOVA with Sidak's multiple comparison corrections; p-values in grey denote non-significant differences.

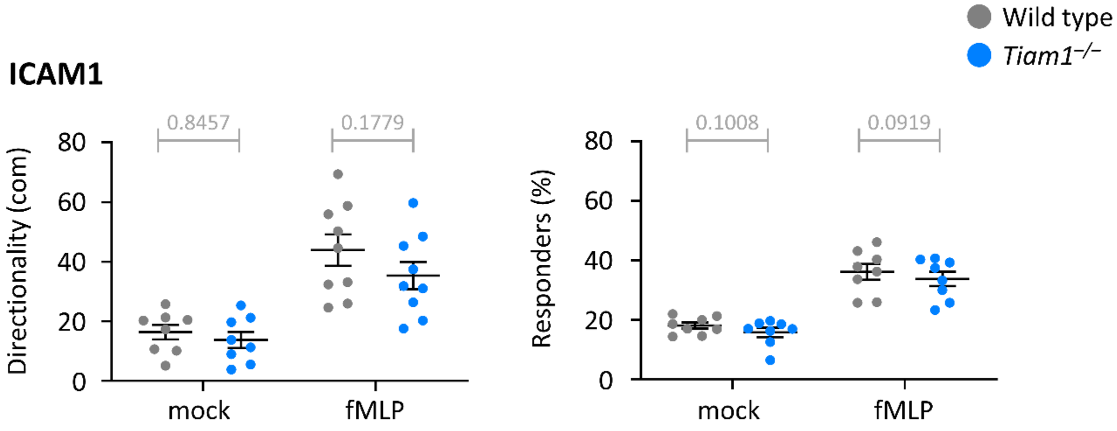

Supplementary Figure 4. Tiam1 does not control directional sensing or the propensity of neutrophils to migrate on ICAM1. Wild type (grey symbols) and *Tiam1*$^{-/-}$ (blue symbols) neutrophils were primed with 50 ng/ml GM-CSF, 20 ng/ml TNFα for 45 min at 37˚C, plated in an ICAM1-coated ibidi chamber (µ-slide VI 0.4), and their migration in a chemoattractant gradient with 10 µM fMLP as the highest concentration (fMLP) or in buffer (mock) was imaged for 20 min. Cells were tracked in the steepest area of the gradient. Data were analysed for directionality of migration and the proportion of cells that migrate using the 'chemotaxis and migration' plugin of Fiji. Data are mean ± SEM of 9 independent experiments, the same as those shown in Figure 5C; each dot is the mean of one experiment. Statistics in are two-way ANOVA with Sidak's multiple comparisons test; p-values in grey are non-significant.

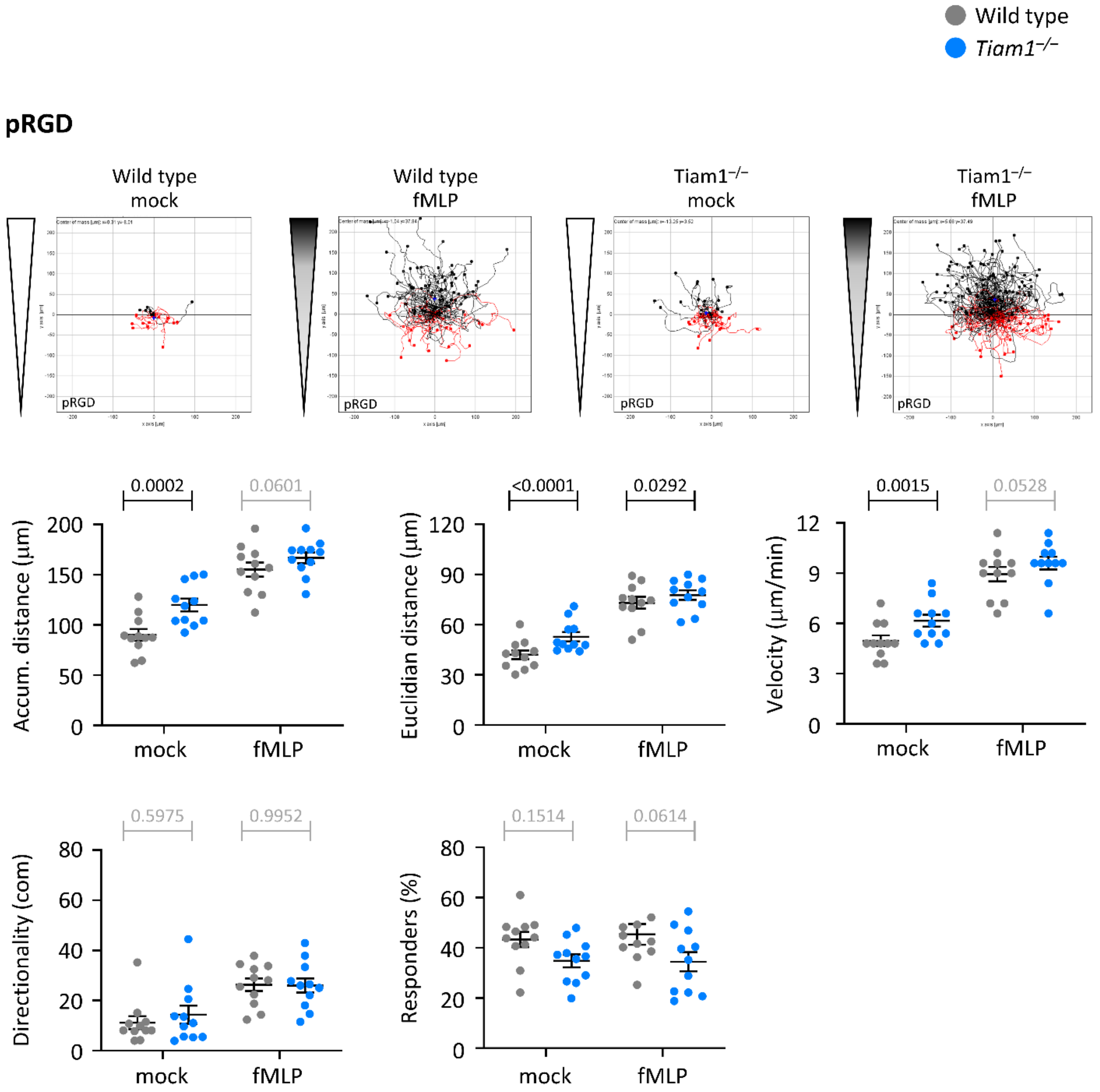

Supplementary Figure 5. Tiam1 limits random neutrophil migration on pRGD. ibidi chamber migration. Wild type (grey symbols) and *Tiam1*$^{-/-}$ (blue symbols) neutrophils were primed with 50 ng/ml GM-CSF, 20 ng/ml TNFα for 45 min at 37˚C, plated into an RGD-coated ibidi chamber (µ-slide VI 0.4), and their migration in a chemoattractant gradient with 10 µM fMLP as the highest concentration (shaded wedges, fMLP), or in buffer only (white wedges, mock), was imaged for 20 min. Cells were tracked in the steepest area of the gradient. Tracks shown are from one representative experiment. Tracks were analysed for the indicated parameters using the 'chemotaxis and migration' plugin of Fiji. Data are mean ± SEM of 11 independent experiments; each dot is the mean of one experiment. Statistics in are two-way ANOVA with Sidak's multiple comparisons test; p-values in black denote significant differences, p-values in grey are non-significant.

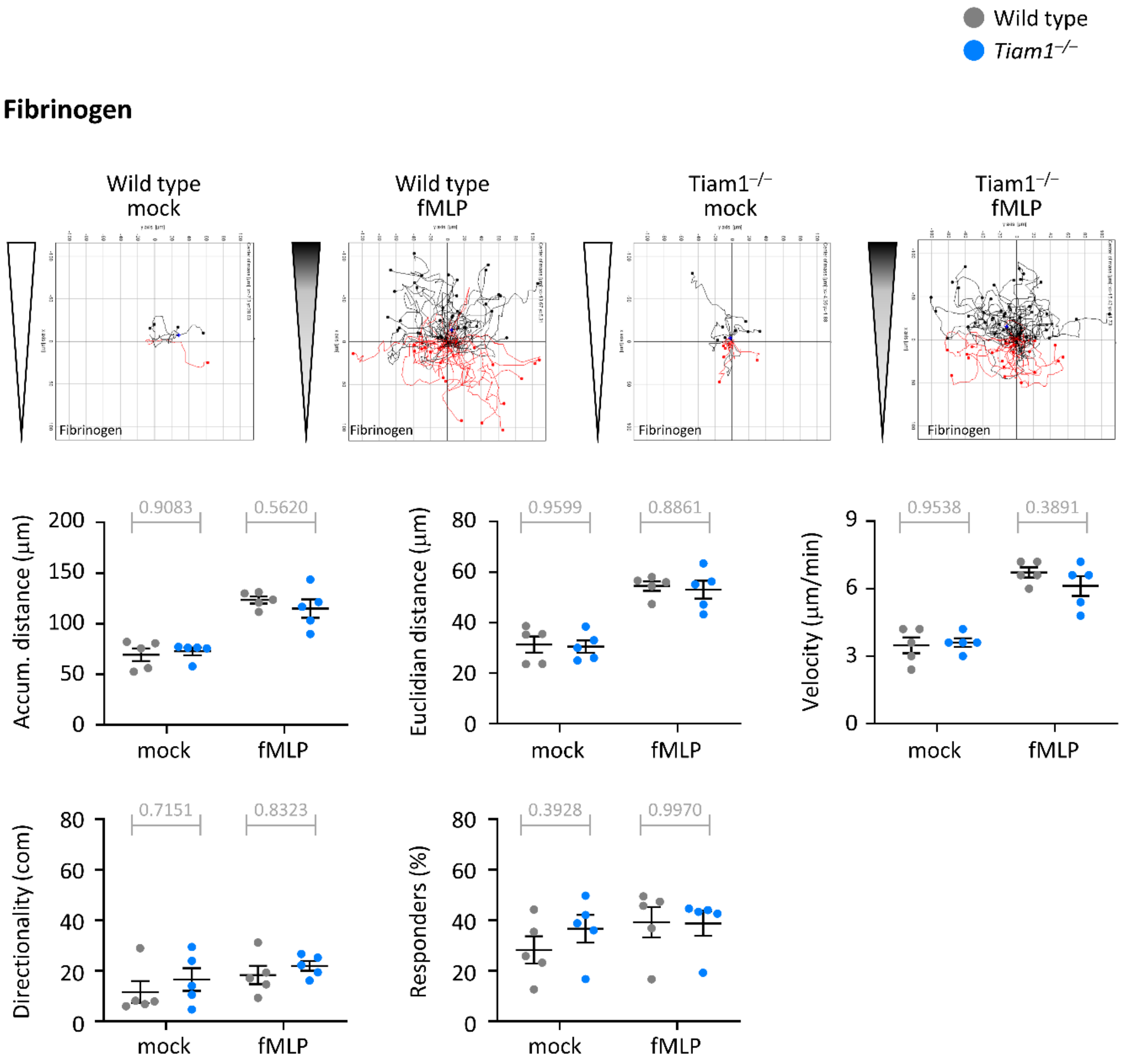

Supplementary Figure 6. Tiam1 does not control neutrophil migration on fibrinogen. Wild type (grey symbols) and *Tiam1*$^{-/-}$ (blue symbols) neutrophils were primed with 50 ng/ml GM-CSF, 20 ng/ml TNFα for 45 min at 37˚C, plated into a fibrinogen-coated ibidi chamber (µ-slide VI 0.4), and their migration in a chemoattractant gradient with 10 µM fMLP as the highest concentration (shaded wedges, fMLP), or in buffer only (white wedges, mock), was imaged for 20 min. Cells were tracked in the steepest area of the gradient. Tracks shown from one representative experiment. Tracks were analysed for the indicated parameters using the 'chemotaxis and migration' plugin of Fiji. Data are mean ± SEM of 5 independent experiments; each dot is the mean of one experiment. Statistics are two-way ANOVA with Sidak's multiple comparisons test; p-values in grey denote non-significance.

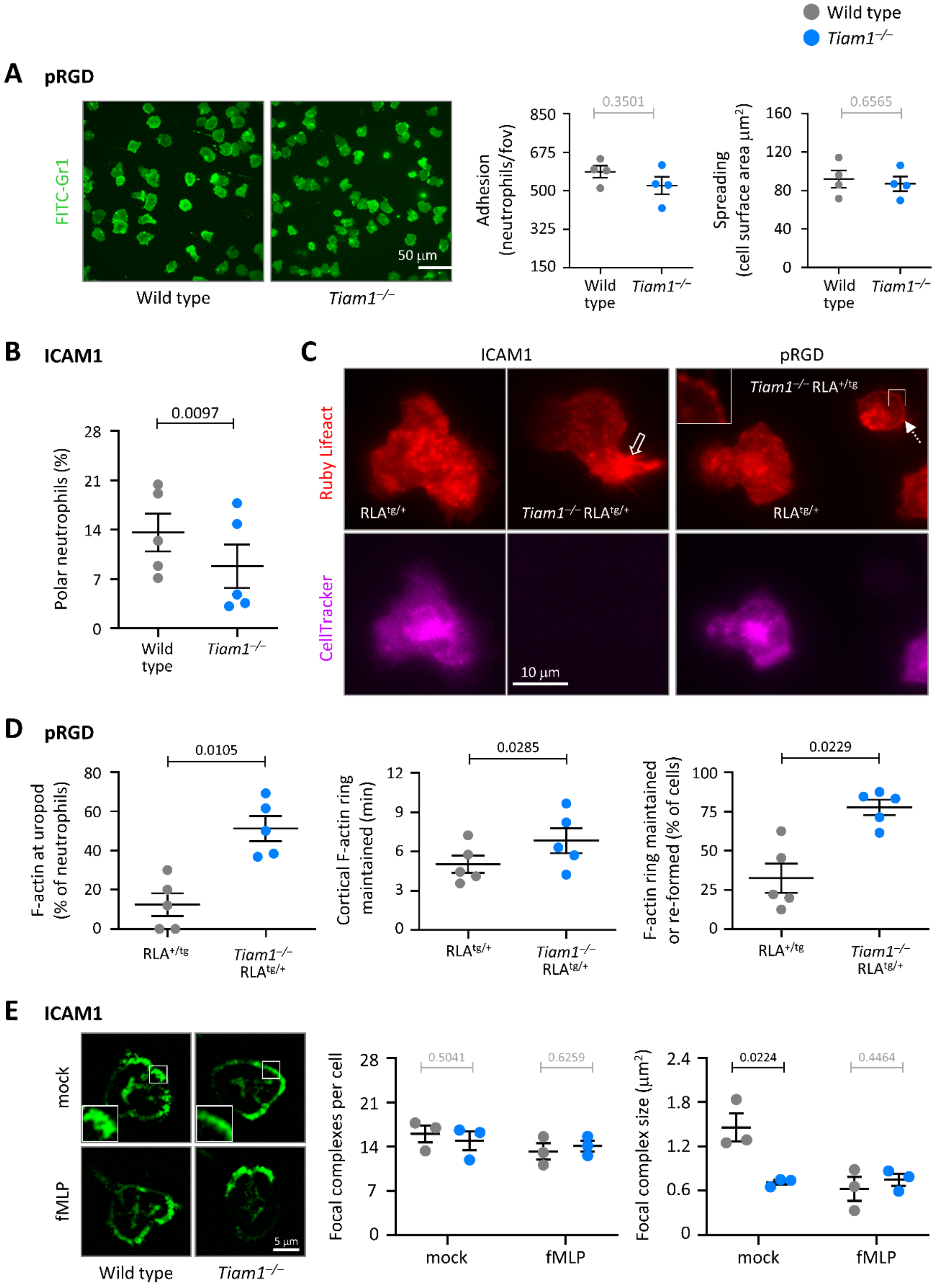

Wild type
Tiam1−/−
A pRGD
FITC-Gr1
50 µm
Wild type
Tiam1−/−
0.3501
Adhesion (neutrophils/fov)
850
675
500
325
150
Wild type
Tiam1−/−
0.6565
Spreading (cell surface area µm²)
160
120
80
40
0
B ICAM1
0.0097
Polar neutrophils (%)
28
21
14
7
0
C
ICAM1
pRGD
Ruby Lifeact
CellTracker
RLAtg/+
Tiam1−/− RLAtg/+
Tiam1−/− RLA+/tg
10 µm
D pRGD
0.0105
F-actin at uropod (% of neutrophils)
80
60
40
20
0
RLA+/tg
Tiam1−/− RLAtg/+
0.0285
Cortical F-actin ring maintained (min)
12
9
6
3
0
0.0229
F-actin ring maintained or re-formed (% of cells)
100
75
50
25
0
E ICAM1
mock
fMLP
5 µm
Wild type
Tiam1−/−
0.5041
0.6259
Focal complexes per cell
28
21
14
7
0
0.0224
0.4464
Focal complex size (µm²)
2.4
1.8
1.2
0.6
0

Supplementary Figure 7. Tiam1 controls neutrophil polarity, F-actin dynamics and focal adhesions. **(A)** Adhesion and spreading. Wild type (grey symbols) and *Tiam1*$^{-/-}$ (blue symbols) neutrophils were prewarmed, plated onto pRGD-coated coverslips for 10 min at 37˚C, fixed, stained with FITC-Gr1 antibody and imaged by widefield fluorescence microscopy. Representative images from one experiment are shown. Cell masks were generated in Fiji. Adhesion was quantified as the number of neutrophils per fov; spreading as the surface area of each cell mask. Data are mean ± SEM of 4 independent experiments, with 27 fov per coverslip and duplicate coverslips per condition assessed in each experiment; each dot is the mean of one experiment. Statistics are paired t-test. **(B)** Neutrophil polarity. Wild type and *Tiam1*$^{-/-}$ neutrophils were allowed to adhere to glass coverslips coated with ICAM1 for 15 min at 37°C, fixed, permeabilised, and stained with phalloidin-Atto 655 and Hoechst 33342. Cell morphology was analysed using widefield fluorescence microscopy, with images blinded prior to analysis. Data are mean ± SEM of 5 independent experiments, with 21-98 cells assessed in each experiment; each dot is the mean of one experiment. Statistics are paired t-test. **(C, D)** F-actin dynamics. RubyLifeact (RLA$^{tg/+}$) and *Tiam1*$^{-/-}$ RLA$^{tg/+}$ neutrophils were primed with 50 ng/ml GM-CSF, 20 ng/ml TNFα for 45 min at 37˚C. RLA$^{tg/+}$ neutrophils were stained with CellTracker and mixed 1:1 with unstained *Tiam1*$^{-/-}$ RLA$^{tg/+}$ neutrophils. Cells were plated onto pRGD or ICAM1, as indicated, in the presence of 0.75 µM fMLP and live-imaged by widefield fluorescence microscopy for 20 min from the moment they started to adhere. (C) Representative images are stills from Supplementary Movies 1 and 2; open arrows denote F-actin at the uropod, stippled arrows the cortical F-actin ring. (D) Quantification. Data are mean ± SEM of 5 independent experiments on pRGD, with movies of 58 RLA$^{tg/+}$ and 72 *Tiam1*$^{-/-}$ RLA$^{tg/+}$ neutrophils analysed; each dot is the mean of one experiment. Statistics in are paired t-test. **(E)** Focal complexes. Wild type and *Tiam1*$^{-/-}$ neutrophils were primed with 50 ng/ml GM-CSF, 20 ng/ml TNFα for 45 min at 37˚C, and allowed to adhere to ICAM1 for 15 min at 37°C in the presence or absence of 0.75 µM fMLP. Cells were fixed, permeabilised, and stained with vinculin antibody and Hoechst 33342. Samples were imaged by TIRF fluorescence microscopy, and images blinded prior to analysis. Representative images; inserts are magnifications of the white squares. The number and size of focal xomplexes were quantified. Data are mean ± SEM of 3 independent experiments, with 49-85 neutrophils analysed per condition; each dot is the mean of one experiment. Statistics are two-way ANOVA with Sidak's multiple comparisons test; p-values in black denote significant differences. (A-E) P-values in black denote significant differences, p-values in grey are non-significant.

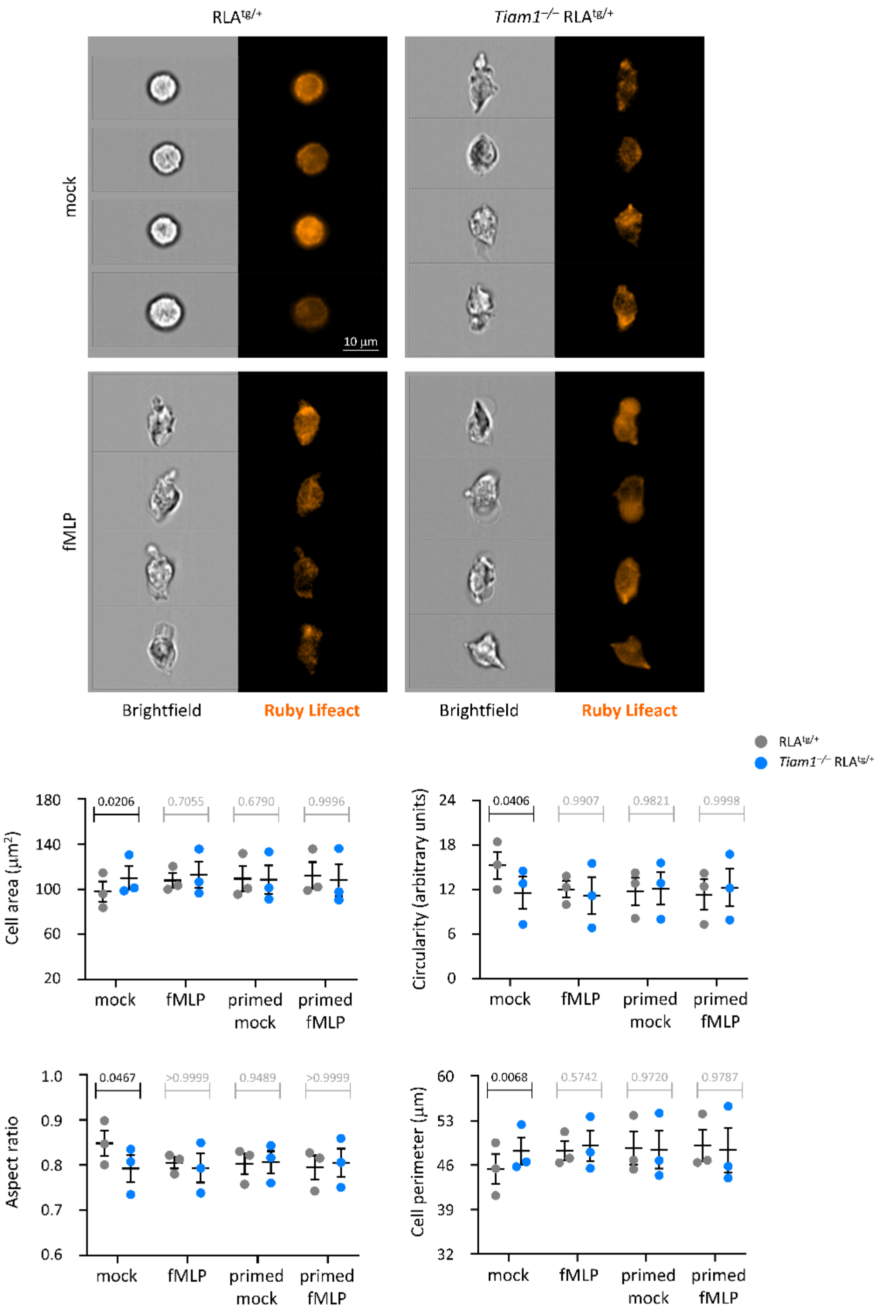

Supplementary Figure 8. Tiam1 maintains the basal morphology of neutrophils in suspension. RLA$^{tg/+}$ (grey symbols) and *Tiam1*$^{-/-}$ RLA$^{tg/+}$ (blue symbols) neutrophils were primed with 50 ng/ml GM-CSF, 20 ng/ml TNFα for 45 min at 37°C, or were kept on ice and prewarmed to 37°C for 3 min prior to the assay. Cells were incubated with 1.5 µM fMLP, or mock-stimulated, for 15 min at 37°C, fixed and analysed on an Imagestream flow cytometer (10,000 events/condition). Ruby Lifeact-positive cells were analysed for area, circularity, aspect ratio (width/length) and perimeter using IDEAS software. Representative images show 4 cells/per condition from one experiment. Data are mean ± SEM of 3 independent experiments; each dot is the mean of one experiment. Statistics are three-way ANOVA with Sidak's multiple comparisons test; p-values in black denote significant differences, p-values in grey are non-significant.

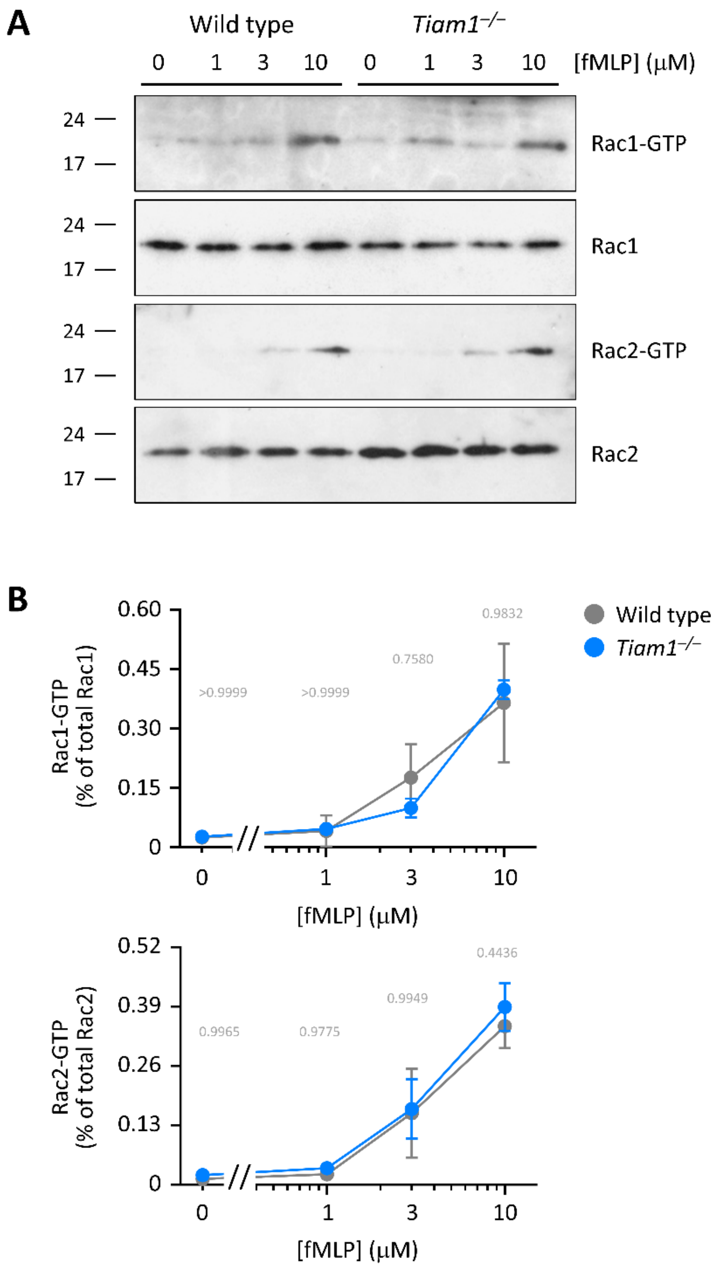

Supplementary Figure 9: Tiam1 does not regulate fMLP-stimulated Rac activity in neutrophils in suspension. Purified wild type (grey symbols) and *Tiam1*$^{-/-}$ (blue symbols) neutrophils in suspension were prewarmed for 3 min at 37°C and then stimulated with the indicated concentrations of fMLP for 10 s. Rac activity (Rac1 and Rac2 GTP-loading) was determined by PAK-CRIB pull down assay. The western blots shown are from one representative experiment; the lower panels show Rac1 and Rac2 levels in 2% of the total lysate for comparison. Quantification was done by densitometry. Data are mean ± SEM of 4 independent experiments. Statistics are two-way ANOVA with Sidak's multiple comparisons test; p-values in grey denote non-significance.

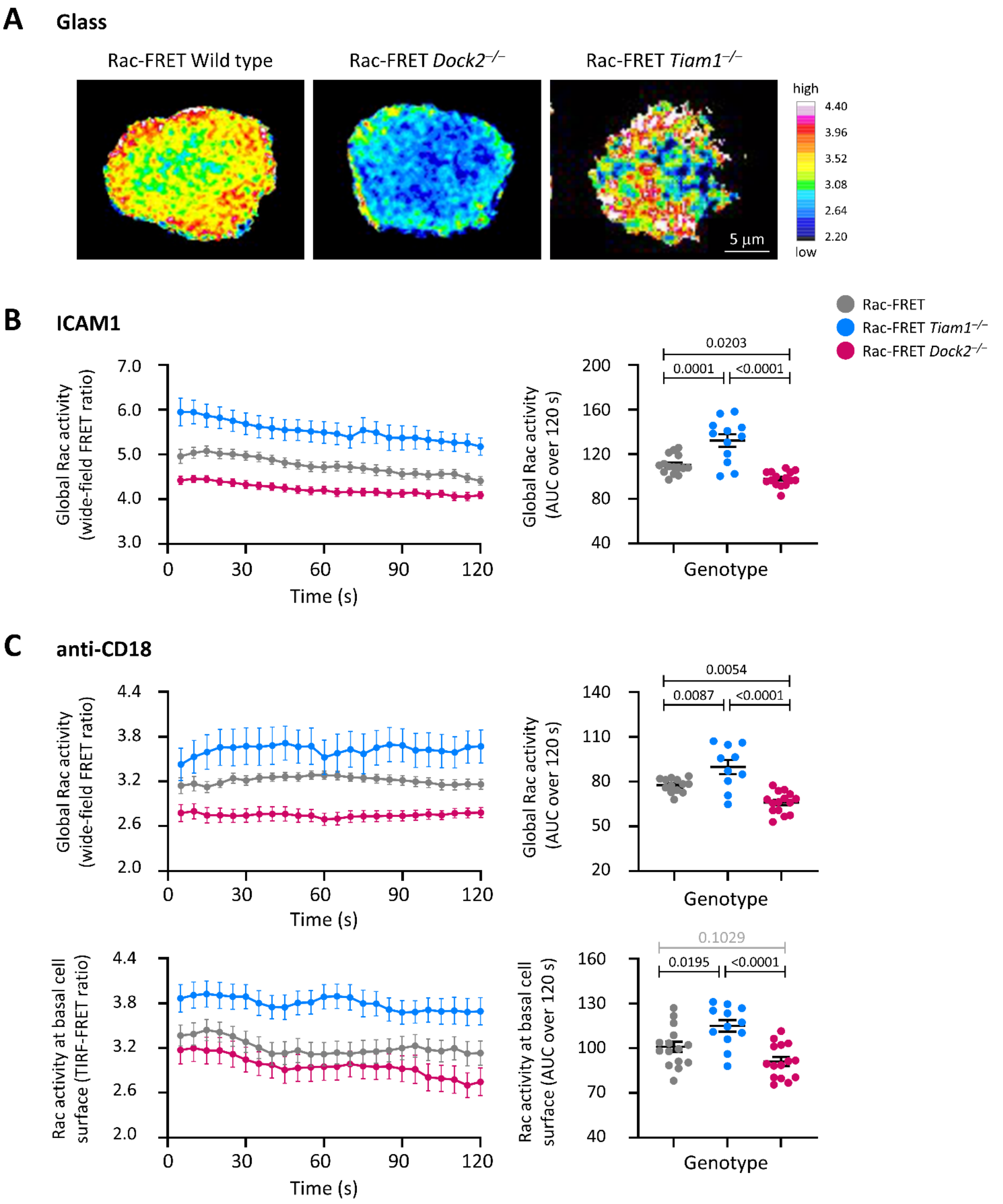
A
Glass
Rac-FRET Wild type
Rac-FRET Dock2−/−
Rac-FRET Tiam1−/−
high
4.40
3.96
3.52
3.08
2.64
2.20
low
5 μm
B
ICAM1
Global Rac activity (wide-field FRET ratio)
Time (s)
Global Rac activity (AUC over 120 s)
Genotype
0.0203
0.0001
<0.0001
Rac-FRET
Rac-FRET Tiam1−/−
Rac-FRET Dock2−/−
C
anti-CD18
Global Rac activity (wide-field FRET ratio)
Time (s)
Global Rac activity (AUC over 120 s)
Genotype
0.0054
0.0087
<0.0001
Rac activity at basal cell surface (TIRF-FRET ratio)
Rac activity at basal cell surface (AUC over 120 s)
0.1029
0.0195
<0.0001

Supplementary Figure 10: Tiam1 limits Rac activity in adherent neutrophils. (A) Rac activity in live neutrophils adhering to glass. Neutrophils from wild type Rac-FRET mice or Rac-FRET mice deficient in Dock2 or Tiam1 were plated onto glass coverslips at 37°C and imaged by live-cell TIRF-FRET microscopy for up to 10 min from the first point of contact with the slide, with frames taken every 5 s. Images are stills of Supplementary Movies 4-6. The pseudocolour scale depicts high Rac activity (FRET ratio) in white/red and low Rac activity in blue. **(B)** Global Rac activity in live neutrophils adhering to ICAM1. Neutrophils from wild type Rac-FET mice (grey symbols), or from Rac-FRET mice deficient in Tiam1 (blue) or Dock2 (pink) were plated onto ICAM1-coated ibidi slides and live-imaged by widefield ratiometric FRET imaging for 2 min at a frame interval of 5 s. Data are global Rac activity (FRET ratio, mean ± SEM) of 12-15 fov per genotype from 3 independent experiments, with direct comparison of all genotypes in each experiment. **(C)** Rac activity globally and at the basal cell surface of live neutrophils adhering to activating anti-CD18 antibody. Neutrophils as in (B) were plated onto ibidi slides coated with activating anti-CD18 antibody and were live-imaged by widefield ratiometric FRET imaging (top) or by ratiometric TIRF FRET imaging (bottom) for 2 min at a frame interval of 5 s. Data are Rac activity (FRET ratio, mean ± SEM) of 10-15 fov per genotype pooled from 3 independent experiments. Left: Rac activity over time. Right: AUC. Statistics in (B, C) are one-way ANOVA with Tukey's multiple comparisons test; p-values in black denote significant differences, p-values in grey are non-significant.

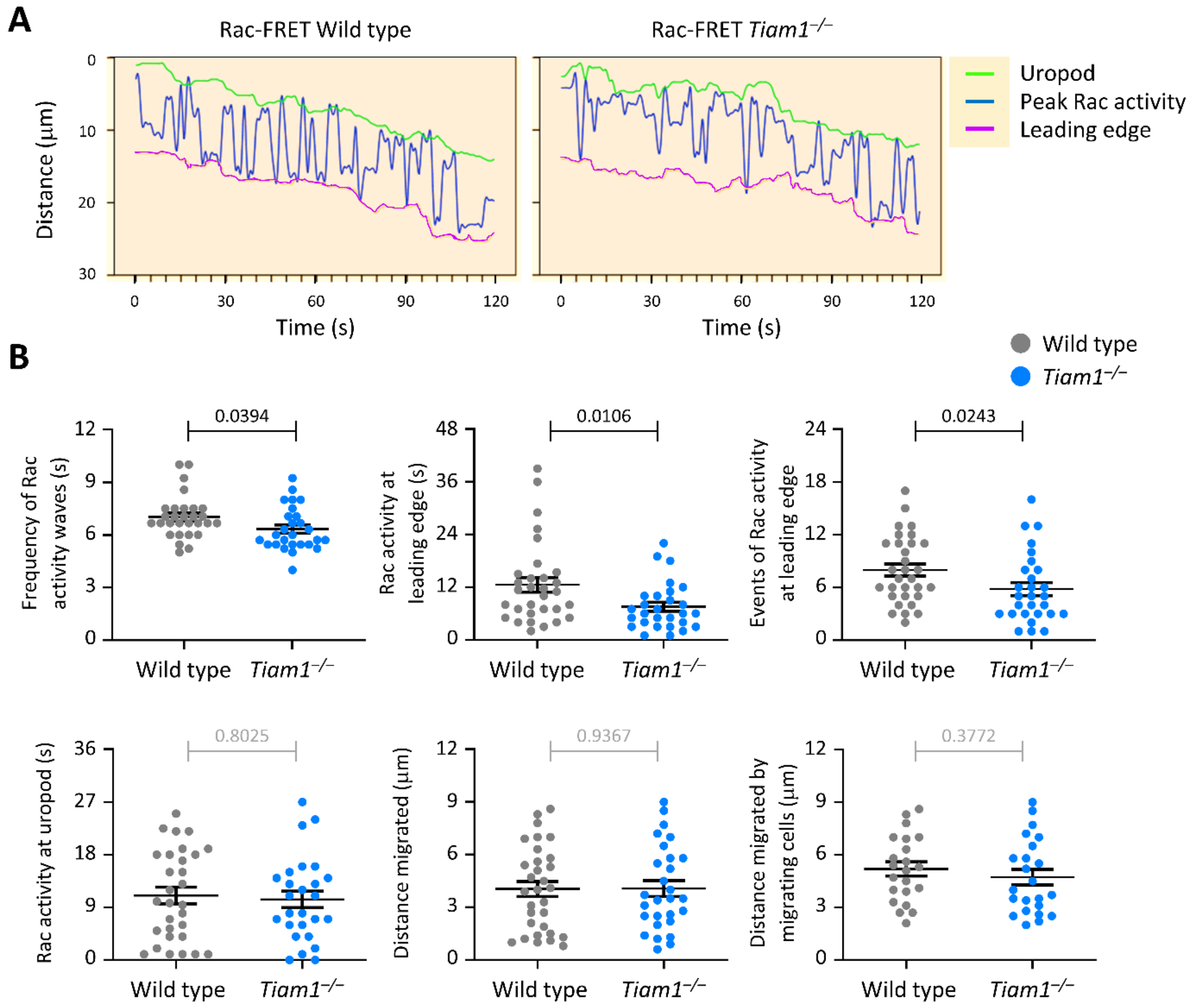

Supplementary Figure 11: Tiam1 localises Rac activity at the leading edge during neutrophil chemotaxis. Neutrophils from wild type Rac-FRET mice (grey symbols) and Rac-FRET *Tiam1*$^{-/-}$ mice (blue symbols) were tested by micropipette chemotaxis assay with fMLP as the chemoattractant. Live-cell ratiometric TIRF-FRET imaging was performed for 2 min at a frame interval of 1 s. Line scans through the central longitudinal axis of the cell were prepared and the localisation of the highest Rac activity (FRET ratio) determined for each frame. **(A)** Representative traces from one experiment, showing the position of peak Rac activity (blue) over time, in relation to the leading edge (pink) and uropod (green). **(B)** Quantification. The frequency (mean ± SEM) of Rac activity oscillations between leading edge and uropod, the time that peak Rac activity was localised within 0.8 µm of the leading edge or uropod, the distance migrated by all cells and by those cells that migrated at least half their body length over the 2 min observation period is plotted. Data are mean ± SEM of 31 wild type Rac-FRET and 28 Rac-FRET *Tiam1*$^{-/-}$ neutrophils from one experiment. Statistics are unpaired t-test; p-values in black denote significant differences, p-values in grey are non-significant.

Supplementary Movie Legends

Supplementary Movie 1. Tiam1 regulates F-actin dynamics in neutrophils adhering to ICAM1. To measure F-actin dynamics during fMLP-stimulated polarisation and chemokinesis in live neutrophils adhering to ICAM1, RubyLifeact ($RLA^{tg/+}$) and *Tiam1*$^{-/-}$ $RLA^{tg/+}$ neutrophils were primed with 50 ng/ml GM-CSF, 20 ng/ml TNFα for 45 min at 37˚C, before $RLA^{tg/+}$ neutrophils were stained with CellTracker and mixed 1:1 with unstained *Tiam1*$^{-/-}$ $RLA^{tg/+}$ neutrophils. Cells were plated onto ICAM1 in the presence of 0.75 µM fMLP and live-imaged by widefield fluorescence microscopy for 20 min from the moment they started to adhere. The representative movies shown are from 1 of 6 independent experiments. The quantification of these experiments is shown in Figure 6C, with movies of 90 $RLA^{tg/+}$ and 131 *Tiam1*$^{-/-}$ $RLA^{tg/+}$ neutrophils analysed in total.

Supplementary Movie 2. Tiam1 regulates F-actin dynamics in neutrophils adhering to pRGD. To measure F-actin dynamics during fMLP-stimulated polarisation and chemokinesis in live neutrophils adhering to pRGD, RubyLifeact ($RLA^{tg/+}$) neutrophils were stained with CellTracker 640 (purple) and mixed 1:1 with unstained *Tiam1*$^{-/-}$ $RLA^{tg/+}$ neutrophils. Cells were plated onto pRGD in the presence of 0.75 µM fMLP and live-imaged by widefield fluorescence microscopy for 20 min from the moment they started to adhere. The representative movies shown are from 1 of 5 independent experiments. The quantification of these experiments is shown in Supplementary Figure 7D, with movies of 58 $RLA^{tg/+}$ and 72 *Tiam1*$^{-/-}$ $RLA^{tg/+}$ neutrophils analysed in total.

Supplementary Movie 3. Tiam1 limits Rac activity at the basal surface of live neutrophils adhering to ICAM1. Neutrophils from wild type Rac-FET mice or from Rac-FRET mice deficient in Tiam1, Dock2 or Prex1/Vav1 were primed with 50 ng/ml GM-CSF, 20 ng/ml TNFα for 45 min at 37˚C and plated onto ICAM1-coated ibidi slides in the presence of 0.75 µM fMLP for 60 min before being live-imaged by ratiometric TIRF-FRET imaging for 2 min at a frame interval of 5 s. This representative movie is from one of 3 independent experiments. Wild type Rac-FET neutrophils (left), Rac-FRET *Tiam1*$^{-/-}$ (second), Rac-FET *Dock2*$^{-/-}$ (third), and Rac-FET *Prex1*$^{-/-}$ *Vav1*$^{-/-}$ (right) cells were directly compared in each experiment. The pseudo-colour scale depicts high Rac activity (high FRET ratio) at the basal cell surface in white/red and low Rac activity in blue. The quantification is shown in Figure 8B. The scale bar is 5 μm

Supplementary Movie 4. Rac activity during the adhesion, spreading and polarisation of wild type Rac-FRET neutrophils on glass. Rac-FRET wild type neutrophils were plated onto glass coverslips and imaged by live-cell TIRF-FRET microscopy from the first point of contact with the slide for 10 min with a frame interval of 5 s to observe Rac activity during adhesion, spreading and polarisation. The movie shown is representative of 8 independent experiments. The pseudo-colouring depicts high Rac activity (high FRET ratio) in white/red and low Rac activity in blue.

Supplementary Movie 5. Rac activity is reduced in Rac-FRET *Dock2*$^{-/-}$ neutrophils adhering to glass. Rac-FRET *Dock2*$^{-/-}$ neutrophils were treated and Rac activity was imaged as for the Rac-FRET wild type neutrophil in Supplementary Movie 4 from the same experiment. The movie is representative of 6 independent experiments. The pseudo-colouring depicts high Rac activity (high FRET ratio) in white/red and low Rac activity in blue.

Supplementary Movie 6. Rac activity is increased in Rac-FRET *Tiam1*$^{-/-}$ neutrophils adhering to glass. Rac-FRET wild type (left), *Tiam1*$^{-/-}$ (middle), and *Dock2*$^{-/-}$ (right) neutrophils were treated and Rac activity was imaged by TIRF-FRET imaging (bottom) as in Supplementary Movie 4 from the same experiment, with brightfield imaging (top) in parallel to visualise the whole cell. The movie is representative of 6 independent experiments. The pseudo-colouring depicts high Rac activity (high FRET ratio) in white/red and low Rac activity in blue.
